# Risk of post-polypectomy bleeding and thromboembolic events during colonoscopy in patients on continued or interrupted antiplatelet therapy: a pooled analysis

**DOI:** 10.3389/fphar.2025.1660871

**Published:** 2025-10-27

**Authors:** Nan-Nan Shen, Jun-Hong Ma, Hua Qian, Yong-Ping Fu

**Affiliations:** ^1^ Department of Pharmacy, Affiliated Hospital of Shaoxing University, Shaoxing, Zhejiang, China; ^2^ Endoscopic Center, Affiliated Hospital of Shaoxing University, Shaoxing, Zhejiang, China; ^3^ Department of Cardiology, Affiliated Hospital of Shaoxing University, Shaoxing, Zhejiang, China

**Keywords:** antiplatelet, bleeding, thromboembolism, polypectomy, colonoscopy

## Abstract

**Background:**

It remains uncertain whether antiplatelet therapy is associated with an increased risk of bleeding in patients undergoing colonoscopic post-polypectomy. Our objective was to compare the incidence of post-polypectomy bleeding and thromboembolic events among patients receiving uninterrupted and interrupted antiplatelet therapy.

**Methods:**

We conducted a comprehensive search of PubMed, MEDLINE, and Cochrane library databases up until March 2024 to identify relevant studies. The primary outcome was the incidence of bleeding events in patients undergoing colonoscopy with polypectomy while continuing or interrupting antiplatelet therapy. Additionally, we assessed the occurrence rate of thromboembolic events as a secondary outcome measure.

**Results:**

Twenty two studies, encompassing 95,107 patients receiving antiplatelet therapy, met the inclusion criteria. Overall, the pooled incidence of colonoscopic post-polypectomy bleeding were 2.40% for patients on uninterrupted clopidogrel, and 2.20% for those on interrupted clopidogrel therapy. Subgroup analysis revealed that older patients on clopidogrel therapy had a higher risk in both uninterrupted (4.60% vs. 1.40%) and interrupted (3.00% vs. 1.50%) treatment regimens compared to younger patients. The incidence of post-procedural bleeding for continued and interrupted aspirin was 1.70% and 1.40%, respectively. Similarly, older individuals on uninterrupted aspirin therapy exhibited a higher risk of bleeding with an incidence rate of 2.50% compared to younger individuals with an incidence rate of l.00%. Among all the regions, the European population on uninterrupted aspirin therapy demonstrated the highest bleeding incidence at 7.20%. Furthermore, thromboembolic events were more prevalent in patients on interrupted clopidogrel than those did not interrupt clopidogrel therapy.

**Conclusion:**

Uninterrupted antiplatelet therapy in elderly patients increases the risk of post-polypectomy bleeding, while the potential elevated risk of thromboembolic events resulting from discontinuation should not be ignored. Especially for high-risk patients, endoscopists must carefully weigh the risk of bleeding and adverse cardiovascular events when deciding whether to interrupt or continue antiplatelet therapy.

## Introduction

The colorectal cancer represents a significant global public health burden, ranking as the third most common malignancy in both incidence and mortality (Sung et al., 2021). Consequently, mitigating its lethal impact is imperative. With the rapid evolution of early screening for colorectal carcinoma, colonoscopic procedures are becoming increasingly popular due to their potential effectiveness in reducing the incidence and mortality of colorectal cancer (Zauber et al., 2012; Amato et al., 2016). However, it is complicated that the common complication is postoperative bleeding, particularly among patients prescribed antiplatelet medications (Yanagisawa et al., 2018). Antiplatelet agents, including aspirin and P2Y12 inhibitors such as clopidogrel, prasugrel, ticagrelor, and cilostazol, are commonly used for thrombosis prevention in cardiovascular and cerebrovascular diseases. Hemorrhage is a major complication associated with antiplatelet therapy. Patients who take antiplatelet agents face a higher bleeding risk compared to those who are not on antiplatelet agents (Pig et al., 2018). It is reported that clopidogrel is associated with an increased the risk of post-polypectomy bleeding (PPB), but aspirin does not (Gandhi et al., 2013). Therefore, ensuring safe conditions during colonoscopy for patients on antiplatelet therapy has become increasingly important (Kim et al., 2019). Nevertheless, the optimal management of antiplatelet therapy around colonoscopy, whether to continue or discontinue, remains uncertain.

The management of antiplatelet therapy in patients undergoing colonoscopy has been debated for several years. However, the available data on whether antiplatelet agents increase bleeding risk and whether cessation is necessary before polypectomy are still limited and conflicting (Manocha et al., 2012; Pan et al., 2012). Current guidelines have emerged regarding the management of antiplatelet therapy for patients undergoing colonoscopy. American guidelines suggest interrupting P2Y12i before colonoscopy in patients with low cardiovascular risk, while continuing therapy in those with high cardiovascular risk, but aspirin should not be interrupted (Committee et al., 2016). European guidelines recommend interrupting P2Y12i 7 days prior to colonoscopy in patients with low cardiovascular risk, whereas consensus among cardiologists is suggested for those at high cardiovascular risk (Veitch et al., 2021a). Nevertheless, the quality of evidence supporting these recommendations ranges from moderate to low. Furthermore, the clinical practice regarding the management of antithrombotics vary widely among colonoscopy clinicians (Kadakia et al., 1996). We must balance the risk of post-polypectomy bleeding against the thromboembolic risk of interrupting antiplatelet therapy (Telford and Abraham, 2022). The proper antiplatelet management around polypectomy should minimize post-procedural bleeding risk bleeding and thromboembolic risks. Therefore, there is an urgent need for high-quality evidence to guide its optimal management.

We performed this systematic review to assess the risks of post-polypectomy bleeding and thromboembolic events in patients receiving uninterrupted antiplatelet therapy compared to those receiving interrupted antiplatelet therapy. Additionally, we aimed to identify potential sources of risk based on patient age, sample size, and geographical region. By synthesizing the available evidence, our objective was to highlight any knowledge gaps and provide direction for future research.

## Methods

This study was performed in accordance with the Systematic Reviews and Meta- analyses (PRISMA) guidelines using a predetermined protocol (PROSPERO: CRD 42024544924).

### Search strategy

A comprehensive electronic search, without any language restriction, was independently performed by two investigators. Studies were identified using the following database: PubMed, Embase, and Cochrane Library for articles published before March 2024. The search strategy is summarized in Supplementary Table S1. Two investigators independently assessed titles and abstracts for eligibility, after removing duplicate records and excluding irrelevant studies based on title and abstract, the eligibility of selected articles from the full text was further reviewed by two authors. Moreover, a manual review of references cited in the selected articles was performed to identify other potentially relevant studies. Discrepancies were resolved by consensus through consultation with a third consultant, referring to the original inclusion and exclusion criteria.

### Selection of studies

The Studies were selected based on the following criteria:

Inclusion criteria: 1) Prospective and retrospective studies without language restriction; 2) Studies that included patients receiving uninterrupted or interrupted antiplatelet therapy undergoing colonoscopy with polypectomy; 3) Antiplatelet agents referred to aspirin or P2Y12 inhibitors (including clopidogrel, prasugrel and ticagrelor, and cilostazol); 4) Studies investigating bleeding or thromboembolic events after polypectomy.

Exclusion criteria: 1) Incomplete outcome data for patients receiving antiplatelet agents; 2) Studies evaluating bleeding or thromboembolic events without discontinuation or continuation of antiplatelet therapy; 3) Patients undergoing colonoscopy without polypectomy.

### Data extraction

The following data variables were independently extracted from the eligible studies: title, first author’s name, publication year, region or country, patients characteristics, sample size, study design, type of antiplatelet agents used, primary and secondary outcomes. All data were recorded as originally stated or after appropriate calculations. In case of missing data, attempts were made to contact the corresponding author for essential information. Additionally, all data extraction was performed independently by two reviewers and compared at the end to minimize selection bias. A third reviewer reviewed the database and any conflicts were resolved through consensus.

### Quality assessment

The risk bias of included studies was independently assessed by two investigators using the modified version of Newcastle-Ottawa Scale (NOS), comprising the following domains: representativeness of sample population, sample size, participation rate, outcome assessment, and analytical methods to control for bias (Cota et al., 2013). Each item could receive a maximum score of 2 points, with a cumulative score >7 indicating an acceptable level of bias (Supplementary Table S2). Any significant conflicts were resolved through consensus or consultation with a senior author if necessary.

### Outcome of interest

The primary outcome of this study was to assess the occurrence rate of delayed post-polypectomy (PPB) bleeding in patients on continuous or interrupted antiplatelet treatment. PPB was defined as a rectal bleeding occurring within 30 days after polypectomy, characterized by overt haemorrhage or decrease in haemoglobin levels of at least 2 g/dL. The secondary outcome was the occurrence of thromboembolic events occurring within 30 days post-colonoscopy were primarily ascertained through clinical diagnosis, with imaging or diagnostic confirmation not being mandatory. They mainly included acute myocardial infarction (AMI), deep vein thrombosis, pulmonary embolism, ischemic stroke, and transient ischemic attack. Subgroup analyses were conducted based on classification of antiplatelet agents, mean age, sample size, and geographical regions.

### Statistical analyses

The outcomes were presented as the pooled rate with a corresponding 95% confidence interval (CI). A random-effects model was performed. Heterogeneity among studies was assessed using *I* ^2^ statistic, with *I* ^2^ > 50% indicating significant heterogeneity (Lima et al., 2025). Subgroup analyses were conducted based on mean age (old and young), sample size (>500 and <500) and different regions (Asian, North American, and European) to investigate the source of heterogeneity. Interaction analyses was applied for the comparability analysis between groups. Sensitivity analysis was performed by sequentially excluding individual studies to examine the robustness of the results. If data from 10 or more studies are available, potential publication bias will be evaluated using funnel plots and Egger’s regression test (Sterne et al., 2011). Meta-regression analysis was conducted to explore the impact of baseline cofounders (mean age, female ratio, HF, HBP, DM, etc.) on the outcomes. Results will be considered statistically significant at a P-value less than 0.05 level. All statistical analyses were conducted using STATA version 13.0 (Statacorp, College Station, Texas, United States).

## Results

### Study search and selection

**FIGURE 1:**
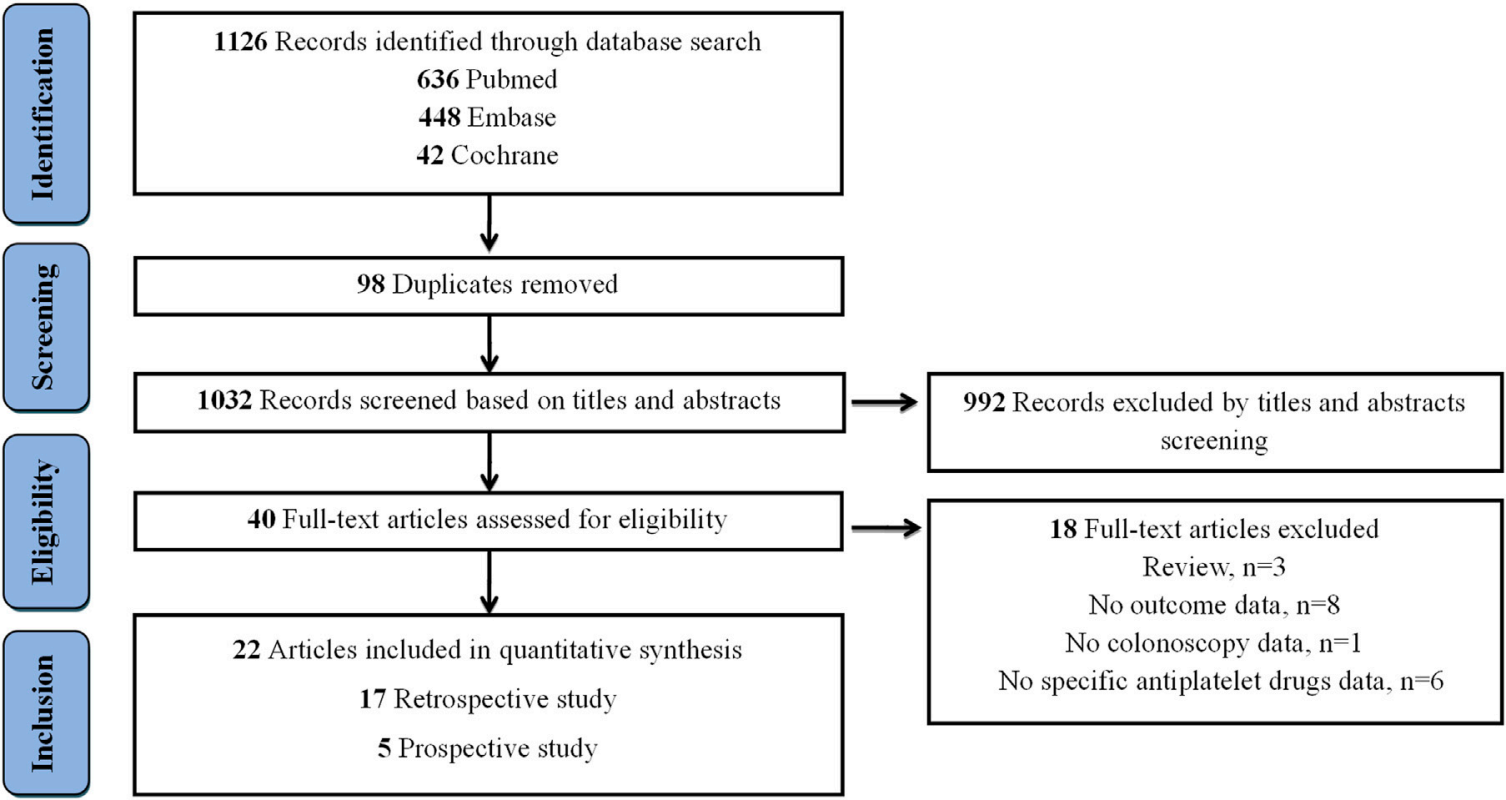
Flow diagram for the selection of eligible studies.

The flow diagram, which presents the results of literature search, is depicted in Figure 1. A total of 1,126 records were retrieved from Pubmed (n = 636), Embase (n = 448), and Cochrane Library (n = 42). After removing 98 duplicated records, an additional exclusion of 1,032 records was performed based on their titles and abstracts. Among 40 full-text articles assessed for eligibility, 18 articles were subsequently excluded, with the following specific exclusion criteria applied: 3 were review articles, 8 lacked relevant outcome data, 1 did not contain colonoscopy data, and 6 failed to provide detailed information on specific antiplatelet medications (Supplementary Table S3). Ultimately, 22 articles were included for quantitative synthesis.

### Study characteristics

The characteristics of twenty-two included studies were summarized in Table 1. Five studies applied a retrospective design, while seventeen utilized a prospective design. Among these studies, thirteen were conducted in Asia (four in China, seven in Japan, one in Turkey, and one in Korea), seven were performed in North America (all from the United States), two originated from Europe (both from Italy). Ten articles assessed PPB rate among patients undergoing colonoscopic polypectomy with uninterrupted clopidogrel therapy, while twelve studies evaluated uninterrupted aspirin therapy. In addition, five studies reported interrupted clopidogrel therapy and four reported interrupted aspirin therapy. Only two articles assessed thromboembolic risk among patients on antiplatelet therapy. A total of 95,107 individuals receiving antiplatelet therapy participated, with sample sizes ranging from 123 to 20,636.

**TABLE 1:** Detailed characteristics of the included studies.

Study	Year	Country	Study design	Data source	Procedure	n
Yousfi M et al.	2004	USA	Retrospective	Mayo Clinic Scottsdale and Rochester	Polypectomy	20,636
Friedland et al.	2009	USA	Retrospective	Veterans Affairs Palo Alto Healthcare System	Polypectomy	123
Singh et al.	2010	USA	Retrospective	Veterans Affairs Medical Center	Polypectomy	1,385
Feagins et al.	2011	USA	Retrospective	Dallas Veterans Affairs Medical Center	Polypectomy	1967
Pan et al.	2012	Italy	Retrospective	Dunedin Hospital	Polypectomy	493
Feagins et al.	2013	USA	Prospective	Veterans Affairs North Texas Healthcare System	Polypectomy	516
Amato et al.	2016	Italy	Prospective	18 endoscopy centres of Lombardy	Polypectomy	2,692
Lin et al.	2018	USA	Retrospective	Veterans Affairs hospital	Polypectomy	20,374
Makino et al.	2018	Japan	Retrospective	Showa Inan General Hospital	Polypectomy	172
Matsumoto et al.	2018	Japan	Retrospective	Sapporo Medical Center	Polypectomy	1,003
Chan et al.	2019	Hong Kong, China	Prospective	Prince of Wales Hospital of The Chinese University	Polypectomy	387
Kishida et al.	2019	Japan	Retrospective	Shizuoka Cancer Center	Polypectomy	6,382
Yu et al.	2019	USA	Retrospective	Clinformatics Data Mart Database	Polypectomy/EMR	11,504
Kishino et al.	2020	Japan	Retrospective	Saku Central Hospital Advanced Care Center	Polypectomy	1930
Yao et al.	2020	Taiwan, China	Retrospective	Kaohsiung Chang Gung Memorial Hospital	Polypectomy	497
Bozkurt et al.	2021	Turkey	Prospective	Tertiary-level public cardiovascular hospital in Istanbul	Polypectomy	119
Kim et al.	2021	Korea	Retrospective	Asan Medical Center	Polypectomy	401
Yabe et al.	2021	Japan	Prospective	Showa Inan General Hospital	Polypectomy	13,017
Yan et al.	2021	China	Retrospective	Beijing Anzhen Hospital	Polypectomy	2,710
Aizawa et al.	2022	Japan	Retrospective	Aizu Medical Center	Polypectomy	2,152
Hayasaka et al.	2023	Japan	Retrospective	Motorman Hospital	Polypectomy	427
Li et al.	2023	Hong Kong, China	Retrospective	Queen Mary Hospital of Hong Kong	Polypectomy	6,220

USA, United States of America; EMR, endoscopic mucosal resection.

### Patient characteristics and quality assessment

The clinical characteristics of patients included in the studies were summarized in Supplementary Table S4. The median age was 65.37 years, with a female representation of 58.31%, and BMI value of 28.85. The cardiovascular comorbidities included hypertension, diabetes mellitus, heart failure, and atrial fibrillation. The risk of bias of articles was summarized in Supplementary Table S5. All studies demonstrated moderate to high quality scores ranging from 6 to 9.

### Post-polypectomy bleeding (PPB) in patients on P2Y12i

**FIGURE 2:**
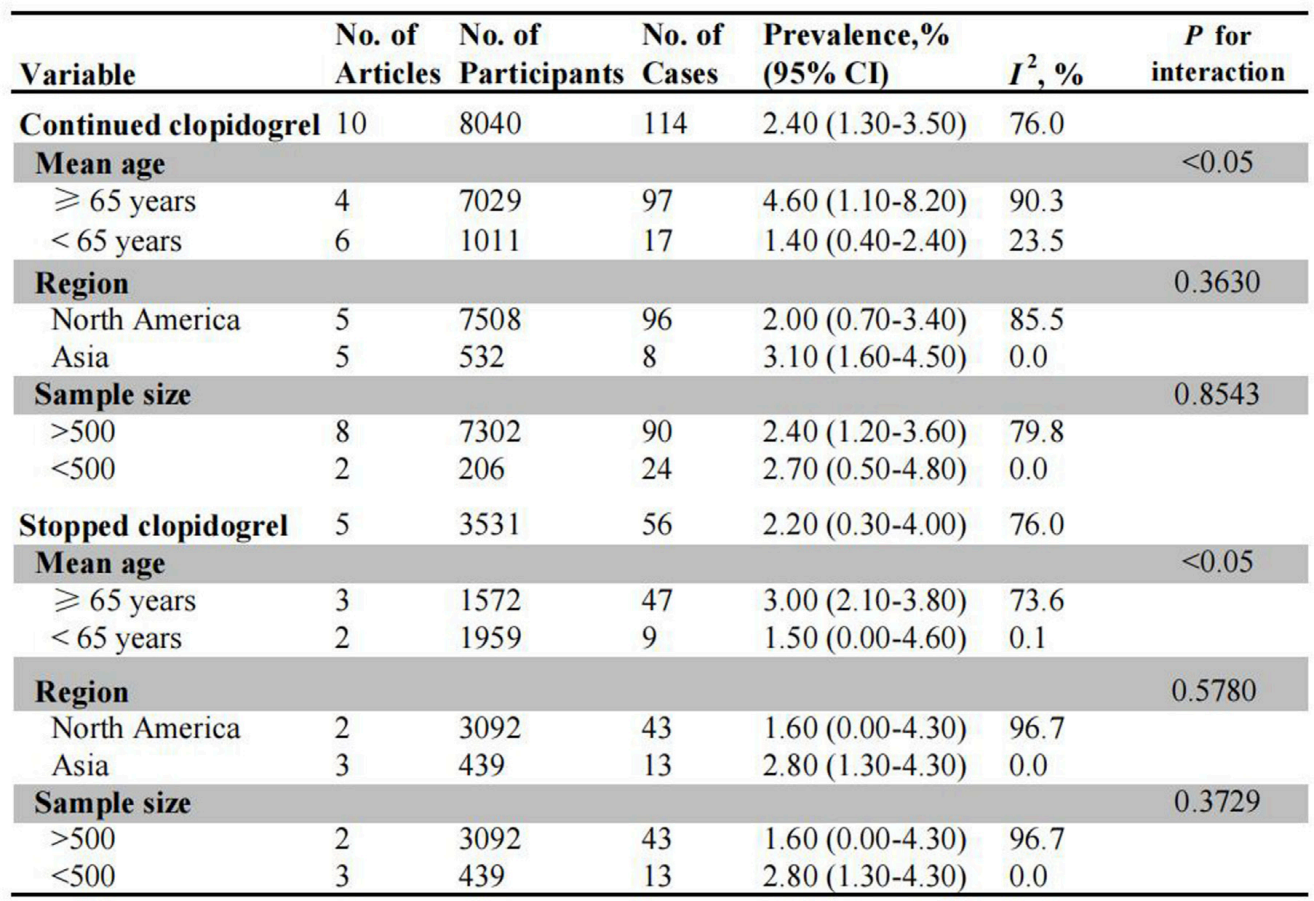
Pooled incidence of bleeding in patients undergoing colonoscopy on uninterrupted and interrupted clopidogrel. No.: number of included studies.

The incidence of PPB in patients receiving uninterrupted and interrupted clopidogrel therapy was summarized in Figure 2. A total of ten studies evaluated the risk of PPB in patients on uninterrupted clopidogrel monotherapy. The pooled incidence of PPB during continued clopidogrel therapy was 2.40% (95% CI: 1.30%–3.50%) (Supplementary Figure S1). Subgroup analysis revealed a higher PPB risk in older age patients (4.60%, 95% CI: 1.10%–8.20%) compared to younger age patients (1.40%, 95% CI: 0.40%–2.40%). Nonetheless, no significant differences were observed in subgroup analysis based on different regions (North America: 2.00% vs. Asia: 3.10%), as well as sample size (>500: 2.40% vs. <500: 2.70%) (Supplementary Figures S2–S4).

Five observational studies assessed PPB risk in patients on interrupted clopidogrel therapy, with the pooled PPB rate being 2.20% (95% CI: 0.30%–4.00%) (Supplementary Figure S5). For the subgroup analysis, there was no significant difference in the incidence of PPB between age stratified subgroups (3.00% for old patients vs. 1.50% for young patients) (Supplementary Figure S6). Similarly, no significant difference was observed between North America (1.60%, 95% CI: 0.00%–4.30%) and Asia (2.80%, 95% CI: 1.30%–4.30%) in terms of region, as well as in subgroup analysis based on sample size (Supplementary Figures S7, S8).

### Post-polypectomy bleeding (PPB) in patients on aspirin

**FIGURE 3:**
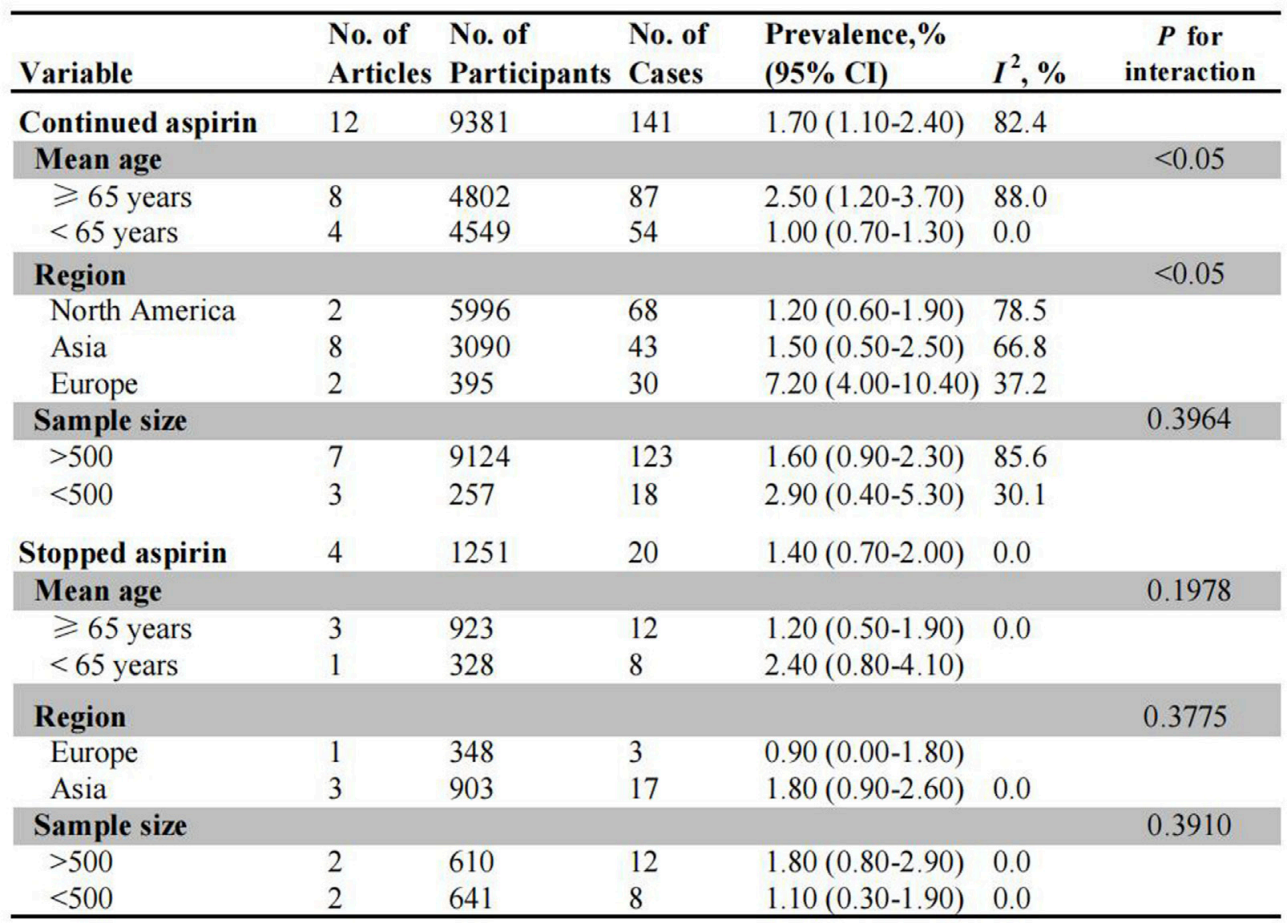
Pooled incidence of bleeding in patients undergoing colonoscopy on uninterrupted and interrupted aspirin. No.: number of included studies.

Twelve studies and four studies evaluated the risk of PPB in patients on uninterrupted and interrupted aspirin monotherapy, respectively (Figure 3). The overall incidence of PPB during uninterrupted aspirin therapy was 1.70% (95% CI: 1.10%–2.40%) (Supplementary Figure S9). In subgroup analysis, older patients exhibited a higher risk of PPB (2.50%, 95% CI: 1.20%–3.70%) than younger patients (1.00%, 95% CI: 0.70%–1.30%) (Supplementary Figure S10). Among different populations, the European population demonstrated the highest risk of PPB (7.20%, 95% CI: 4.00%–10.40%), followed by Asian population (1.50%, 95% CI: 0.50%–2.50%) and North American population (1.20%, 95% CI: 0.60%–1.90%) (Supplementary Figure S11). There was no significant difference in bleeding incidence between studies with larger sample size (1.60%, 95% CI: 0.90%–2.30%) and those with smaller sample sizes (2.90%, 95% CI: 0.40%–5.30%) (Supplementary Figure S12).

Among patients on interrupted aspirin before colonoscopy, the combined incidence of bleeding was 1.40% (95% CI: 0.70%–2.00%) (Supplementary Figure S13). Asian patients exhibited a higher rate of bleeding events (1.80%, 95% CI: 0.90%–2.60%) compared to European patients (0.90%, 95% CI: 0.00%–1.80%), although this difference was not statistically significant (Supplementary Figure S14). No significant differences were observed in the subgroups based on age stratification (≥65 years: 1.20% vs. <65 years: 2.40%) or sample size (>500: 1.80% vs. <500: 1.10%) (Supplementary Figures S15, S16).

### Post-polypectomy thromboembolism (TE) in patients on antiplatelet therapy

**FIGURE 4:**
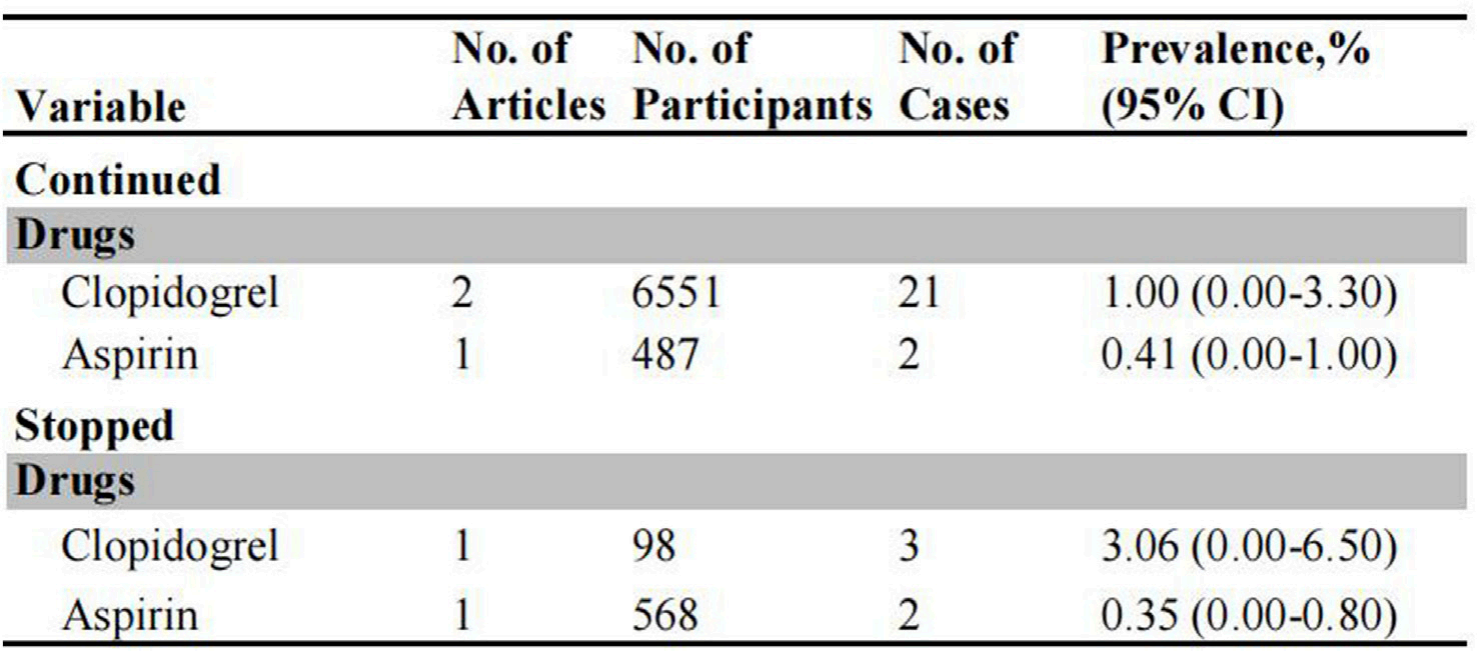
Pooled incidence of thromboembolic events in patients undergoing colonoscopy on uninterrupted and interrupted antiplatelet therapy.

The risk of post-polypectomy TE events was investigated in patients receiving uninterrupted and interrupted antiplatelet agents (Figure 4). The incidence of post-polypectomy TE was significantly higher in patients on interrupted clopidogrel (3.06%, 95% CI: 0.00%–6.50%) compared to those who continued clopidogrel therapy (1.00%, 95% CI: 0.00%–3.30%) (Supplementary Figure S17). There was no significant difference in the risk of TE events between patients receiving uninterrupted aspirin therapy (0.35%, 95% CI: 0.00%–0.80%) and those on interrupted aspirin therapy (0.41%, 95% CI: 0.00%–1.00%). Whereas, since these findings are based on only two studies, the results should be interpreted carefully.

### Sensitivity analysis and meta-regression

Sensitivity analyses of antiplatelet therapy were performed by sequentially removing each study to assess the impact of individual studies on the pooled estimates (Supplementary Figures S6, S7). Subsequent PPB sensitivity analysis yielded consistent findings after removing each individual study. Meta-regression analysis was employed to explore the potential effect of patient characteristics on bleeding incidence and TE events (Supplementary Figure S8). Meta-regression analysis demonstrated no statistically significant correlations between variables and the pooled incidence.

### Publication bias

No potential publication bias was observed in continued clopidogrel and aspirin therapy by qualitative funnel plots as well as Begg’s test and Egger’s test (Supplementary Figures S18, S19). Because of the limited study number in stopped clopidogrel and aspirin therapy (<8 studies), the funnel plot was not performed.

## Discussion

This study comprehensively evaluates the bleeding and thromboembolic risk in patients undergoing colonoscopic polypectomy while receiving uninterrupted or interrupted antiplatelet therapy. Our findings indicate that there is no significant increase in the risk of post-polypectomy bleeding among patients on uninterrupted antiplatelet therapy compared to those on interrupted therapy. However, it is important to underline that elderly patients have an elevated risk of PPB. It should be noted that limited studies provided data on subgroups due to inadequate sample sizes.

Several meta-analyses have evaluated the risk of colonoscopic post-polypectomy bleeding in patients receiving antiplatelet therapy. However, these studies have yielded contradictory conclusions (Gandhi et al., 2013; Pig et al., 2018; Li et al., 2020). Among these studies, only one systematic review assessed the bleeding risk specially in patients on single antiplatelet therapy. Furthermore, none of these studies have systematically assessed thromboembolic risk associated with antiplatelet therapy. Thus, there is still limited data comparing the risk of bleeding and thromboembolism between individuals who discontinue or continue taking antiplatelet agents prior to polypectomy. Unlike previous studies that solely focused on bleeding risk, we simultaneously evaluated the risk of thromboembolism. Although uninterrupted antiplatelet therapy is associated with a high bleeding risk, aspirin withdrawal increased risk of cardiovascular adverse events (Biondi-Zoccai et al., 2006). Additionally, current guidelines regarding interruption or continuation of antiplatelet therapy are mainly based on expert opinion with weak evidence (Abdel et al., 2014). Notably, few therapeutic indications for single antiplatelet therapy are present in current cardiological guidelines (Collet et al., 2021; Ibanez et al., 2017). Henceforth, it is crucial to strike a balance between the risks posed by uninterrupted antiplatelet therapy for PPB and major adverse cardiovascular events before colonoscopic polypectomy. The updated European Society of Gastrointestinal Endoscopy (ESGE) guideline recommends maintaining P2Y12 inhibitors therapy for low-risk procedures (Veitch et al., 2021b). Nevertheless, discontinuation of P2Y12 inhibitors is advised for high-risk procedures. Notably, prior research demonstrate that thromboembolic events may manifest as early as 7 days following clopidogrel withdrawal (Eisenberg et al., 2009). Consequently, the decision to discontinue clopidogrel therapy warrants careful reconsideration, particularly in patients with elevated thrombotic risk.

Clopidogrel, a P2Y12 inhibitor, is widely utilized in the management of patients with coronary syndrome events and stroke (Gerhard-Herman et al., 2017; Kernan et al., 2014). Previous investigations have explored whether temporary discontinuation of clopidogrel should be considered for patients undergoing colonoscopy. However, these studies have yielded conflicting findings regarding increased bleeding risk (Feagins et al., 2013; Singh et al., 2010) or no excessive bleeding risk (Feagins et al., 2011). Some studies have reported a low incidence of post-polypectomy bleeding among patients who continued clopidogrel therapy through the period of polypectomy (Chan et al., 2019; Won et al., 2019). Our results are in line with these studies as we observed a low rate of bleeding and no significant difference in the occurrence of post-polypectomy bleeding between uninterrupted and interrupted subjects. Nevertheless, we observed a significantly increased risk of post-polypectomy bleeding in elderly patients receiving continued clopidogrel treatment. This can primarily be attributed to older individuals tend to be at higher bleeding risk due to comorbid medical conditions and multiple medications which may alter metabolism and excretion of antiplatelet drugs (Abraham, 2020). Additionally, advanced age has been recognized as an important factor predicting post-polypectomy bleeding, and increasing the likelihood of receiving blood transfusions (Sorbi et al., 2000). For elderly patients, therapeutic decision-making regarding P2Y12 inhibitors continuation requires particular caution. Given the inherent complexity of balancing thrombotic and bleeding risks, it should be recommended liaising with the consultant interventional cardiologists about the risk/benefit of discontinuing P2Y12 inhibitors. One study indicated that a greater proportion of patients who continued taking clopidogrel experienced post-polypectomy bleeding (Kishino et al., 2020). However, it is important to underline that post-polypectomy bleeding is rarely life threatening, thrombotic event caused by clopidogrel interruption may be harmful. Therefore, further deliberation about the balance between bleeding risk and cardiovascular thrombotic events when interrupting antiplatelet therapy is essential (Sonneveld et al., 2019; Radaelli et al., 2019).

Aspirin has been widely used in clinical practice for the prevention of thrombosis worldwide. Generally, there is no need to discontinue aspirin prior to polypectomy due to low risk of post-polypectomy bleeding (Yousfi et al., 2004). The Japanese guideline does not recommend discontinuation of aspirin therapy before colonoscopic polypectomy procedures (Fujimoto et al., 2014). A study reported no significant difference in the incidence of post-polypectomy bleeding between patients who continued or stopped aspirin (Yousfi et al., 2004). However, caution should be exercised when removing large colonic polyps (Feagins et al., 2013). It is important to note that advanced age independently increases the risk of bleeding in patients undergoing colonoscopic polypectomy (Committee et al., 2016; Veitch et al., 2021a). Our study is in line with these data, uninterrupted aspirin therapy do not increase bleeding risk compared to interrupted use during colonoscopy, but a high risk of post-polypectomy bleeding was observed in elderly patients. Literature reported that elderly patients over 75 years were more likely to require re-hospitalization within 7 days after colonoscopy (Grossberg et al., 2020), primarily due to their multiple comorbidities such as cardiovascular disease in antithrombotic treatment, especially antiplatelet therapy (Heitman et al., 2009; O'Brien et al., 2019).

Physicians often prioritize the risk of post-polypectomy bleeding due to its immediate occurrence, while stroke may manifest later and potentially go unnoticed by the performing physician during colonoscopy (Alonso-Coello et al., 2015; Doorey et al., 2018). Especially in high-risk patients, thrombosis resulting from temporary cessation of antiplatelet may be fatal. Therefore, a collaborative evaluation of bleeding and thromboembolism risk is essential to determine an appropriate treatment regimen. Current guidelines in the U.S. and Europe recommend withholding clopidogrel for 7 days prior to colonoscopic polypectomy (Committee et al., 2016; Veitch et al., 2016). However, it has been reported that thromboembolic events can occur as early as 7 days after interrupting clopidogrel (Eisenberg et al., 2009). Our findings also indicated an increased incidence of thromboembolic events with interrupted clopidogrel therapy. Therefore, it is important to consider the potential elevated risk of thromboembolic events in patients on uninterrupted antiplatelet therapy. However, existing evidence regarding thromboembolic risk remains limited due to a scarcity of studies. In particular, there is a lack of studies comparing risk of thromboembolism between patients continuing antiplatelet therapy versus those discontinue it. Thus, it is important to generate stronger data concerning bleeding and thromboembolism risks in patients receiving antiplatelet therapy.

Currently, no randomized controlled trials (RCTs) have directly compared bleeding and thromboembolic risks between uninterrupted and interrupted antiplatelet therapy in patients undergoing polypectomy. Furthermore, existing evidence regarding antiplatelet management in this population remains inconclusive (Abdel et al., 2014; Collet et al., 2021). To address this gap, we conducted a systematic review to evaluate these trade-offs in real-world clinical practice. Our findings suggest that continuing antiplatelet therapy may be acceptable, however, elderly patients on uninterrupted regimens demonstrated an elevated bleeding risk. Future studies are warranted to determine optimal management strategy for antiplatelet therapy in this clinical context.

This study provides real-world data on the risk of post-colonoscopy bleeding and thromboembolism with a large sample size, thereby enhancing the reliability of evidence. However, several limitations exist in this study. Firstly, due to the unavailability of RCTs evaluating bleeding and thromboembolic risk on antiplatelet therapy, only observational studies were included, which may undermine the value of extrapolating these results to clinical practice. Although observational studies are often considered methodologically weaker than RCTs due to potential confounding, they are increasingly recognized as complementary (Jarow et al., 2017). By extending the findings to larger, real-world patient populations, observational studies and their meta-analyses provide decision-makers with evidence that is broadly applicable. Secondly, our study solely focused on assessing the risk of delayed bleeding due to its unpredictable nature and potential serious consequences, in contrast to the easily detectable and controllable immediate bleeding following colonoscopic polypectomy. Thirdly, our study assessed bleeding risk among patients on single clopidogrel and aspirin, and not any performed sub-analysis among other antiplatelet agents due to no available data. Fourthly, although risk of post-polypectomy bleeding has been reported to be associated with polyp size, location, and the types of medications (Feagins et al., 2019; Kothari et al., 2019; Aiza et al., 2022), these variables were not considered in our included studies due to data absence. Additionally, given the limited number of patients undergoing polypectomy while on dual antiplatelet therapy, we did not explore these cases, but plan to investigate them in future studies. Finally, the generalizability of our findings in subgroups may be constrained by the limited number of studies.

## Conclusion

In summary, the incidence of colonoscopic post-polypectomy bleeding was comparable between uninterrupted and interrupted antiplatelet users, whereas a higher risk of bleeding was observed in elderly patients on uninterrupted antiplatelet therapy. Patients receiving interrupted clopidogrel had an increased risk of thromboembolism compared to those receiving uninterrupted clopidogrel. Therefore, the continuation of antiplatelet therapy may be acceptable for colonoscopic polypectomy, but caution should be exercised in elderly patients with a high risk of adverse cardiovascular events. In future, randomized controlled studies are necessary to further elucidate the issue surrounding antiplatelet use during polypectomy.

## Data Availability

The original contributions presented in the study are included in the article/Supplementary Material, further inquiries can be directed to the corresponding authors.
