# Supplementary material for: Risk of post-polypectomy bleeding and thromboembolic events during colonoscopy in patients on continued or interrupted antiplatelet therapy: a pooled analysis
Source: Front Pharmacol. 2025 Oct 27;16:1660871. doi: 10.3389/fphar.2025.1660871 (PMC12597905; doi:10.3389/fphar.2025.1660871)
Supplement: Supplementary file 1 [file Supplementaryfile1.docx]

SUPPLEMENTAL FILE

Title: The risk of bleeding and thromboembolic events in patients undergoing colonoscopy on

uninterrupted or interrupted antiplatelet therapy: a pooled analysis

Contents

**Tables**

[eTable 1. Search strategy to identify studies reporting the incidence of bleeding and thromboembolic events in patients undergoing colonoscopy on interrupted or uninterrupted antiplatelet therapy... 3](#_Toc42611083)

[eTable 2. Quality assessment scale 4](#_Toc42611084)

[eTable 3. Excluded studies with reasons 6](#_Toc42611084)

[eTable 4. Detailed demographics and clinical ch](#_Toc42611086)[aracteristics of the included studies 7](#_Toc42611086)

[eTable 5. Quality scores of the included studies 8](#_Toc42611087)

[eTable 6. Sensitivity](#_Toc42611090) [analysis of PPB/TE rate in patients with or without continued clopidogrel therapy. 9](#_Toc42611090)

[eTable 7. Sensitivity analysis of PPB/TE rate in patients with or without continued aspirin therapy. 1](#_Toc42611090)0

[eTable 8. Meta-regression of the risk of bleeding and thromboembolic events in patients undergoing colonoscopy 1](#_Toc42611091)1

**Figures**

[eFigure 1. Pooled prevalence of PPB rate in patients with continued clopidogrel therapy 1](#_Toc42611097)2

[eFigure 2. Pooled prevalence of PPB rate in patients with continued clopidogrel therapy by age 1](#_Toc42611099)2

[eFigure 3. Pooled prevalence of PPB rate in patients with continued clopidogrel therapy by region 1](#_Toc42611099)3

[eFigure 4. Pooled prevalence of PPB rate in patients with continued clopidogrel therapy by sample 1](#_Toc42611099)3

[eFigure 5. Pooled prevalence of PPB rate in patients without continued clopidogrel therapy 1](#_Toc42611098)4

[eFigure 6. Pooled prevalence of PPB rate in patients without continued clopidogrel therapy by age 1](#_Toc42611099)4

[eFigure 7. Pooled prevalence of PPB rate in patients without continued clopidogrel therapy by region 1](#_Toc42611099)5

[eFigure 8. Pooled prevalence of PPB rate in patients without continued clopidogrel therapy by sample 1](#_Toc42611099)5

[eFigure 9. Pooled prevalence of PPB rate in patients with continued aspirin therapy 1](#_Toc42611098)6

[eFigure 10. Pooled prevalence of PPB rate in patients with continued aspirin therapy by age 1](#_Toc42611099)6

[eFigure 11. Pooled prevalence of PPB rate in patients with continued aspirin therapy by region 1](#_Toc42611099)7

[eFigure 12. Pooled prevalence of PPB rate in patients with continued aspirin therapy by sample 1](#_Toc42611099)7

[eFigure 13. Pooled prevalence of PPB rate in patients without continued aspirin therapy 1](#_Toc42611099)8

[eFigure 14. Pooled prevalence of PPB rate in patients without continued aspirin therapy by age 1](#_Toc42611099)8

[eFigure 15. Pooled prevalence of PPB rate in patients without continued aspirin therapy by region 1](#_Toc42611099)9

[eFigure 16. Pooled prevalence of PPB rate in patients without continued aspirin therapy by sample 1](#_Toc42611099)9

[eFigure 17. Pooled prevalence of TE rate in patients with continued clopidogrel therapy 2](#_Toc42611099)0

[eFigure 18. Publication bias of studies on PPB rate in patients with continued clopidogrel therapy 2](#_Toc42611099)0

[eFigure 19. Publication bias of studies on PPB rate in patients with continued aspirin therapy 2](#_Toc42611099)1

[References 22](#_Toc42611100)

# eTable 1. Search strategy to identify studies reporting the incidence of bleeding and thromboembolic events in patients undergoing colonoscopy on interrupted or uninterrupted antiplatelet therapy.

| **Literature databases** | **Search items** | **Items found** |
| --- | --- | --- |
| MEDLINE via Pubmed | #1: “colonoscopy”[MeSH Terms] OR “colonoscopy”[Title/Abstract] OR “polypectomy” [Title/Abstract] OR “ polypectomy ”[Title/Abstract] OR “enteroscopy”[Title/Abstract]  #2 : “clopidogrel”[MeSH Terms] OR “clopidogrel”[Title/Abstract] OR “aspirin”[MeSH Terms] OR “aspirin”[Title/Abstract] OR “ticagrelor ”[MeSH Terms] OR “ticagrelor ”[Title/Abstract] OR “antiplatelet drug” [MeSH Terms] OR “antiplatelet drug”[Title/Abstract]  #1 AND #2 | 636 |
| EMBASE | #1: ‘colonoscopy’/exp OR ‘colonoscopy’:ti,ab,kw OR ‘colonoscopy’: ti,ab,kw OR ‘polypectomy’ /exp OR ‘polypectomy’: ti,ab,kw OR ‘enteroscopy’: ti,ab,kw  #2: ‘clopidogrel’/exp OR ‘clopidogrel’:ti,ab,kw OR ‘aspirin’/exp OR ‘aspirin’: ti,ab,kw OR ‘ticagrelor ’/exp OR ‘ticagrelor ’:ti,ab,kw OR ‘antiplatelet drug’/exp OR ‘antiplatelet drug’: ti,ab,kw  #1 AND #2 | 448 |
| COCHRANE | #1: MeSH descriptor: [colonoscopy] OR colonoscopy: ti,ab,kw OR polypectomy: ti,ab,kw OR polypectomy: ti,ab,kw OR enteroscopy: ti,ab,kw  #2: MeSH descriptor: [clopidogrel] OR clopidogrel: ti,ab,kw OR MeSH descriptor: [aspirin] OR aspirin ti,ab,kw OR MeSH descriptor: [ticagrelor] OR ticagrelor: ti,ab,kw OR MeSH descriptor: [antiplatelet drug] OR antiplatelet drug: ti,ab,kw  #1 AND #2 | 42 |
| Overall |  | 1126 |
| Duplication |  | 98 |

**eTable 2. Quality assessment scale**

| **Bias type** | **Selection**  **(sample population)** | **Selection**  **(sample size)** | **Selection (participation rate)** | **Performance bias (outcome assessment)** | **Performance bias (analytical methods to control for bias)** |
| --- | --- | --- | --- | --- | --- |
| **Low risk**  **(score=2)** | 1) Sample from the general population, not a select group;  2) Consecutive unselected population;  3) Rationale for case and control selection explained. | 1) Sample size calculation performed and adequate. | 1) High response rate (>85%). | 1) Diagnosis using consistent criteria and direct examination. | 1) Analysis appropriate for the type of sample (subgroup analysis/regression etc.) |
| **Moderate risk (score=1)** | 1) Sample selected from large population but selection criteria not defined;  2) Sample selection ambiguous but may be representative;  3) Rationale for cases and controls not explained;  4) Eligibility criteria not explained;  5) Analysis to adjust for sampling strategy bias. | 1) Sample size calculation performed and reasons for not meeting sample size given;  2) Sample size calculation not performed but all eligible persons studied. | 1) Moderate response rate (70-85%). | 1) Assessment from administrative database or register;  2) Assessment from hospital record or interviewer. | 1) Analysis does not account for common adjustment. |
| **High risk (score=0)** | 1) Highly select population making it difficult to generalise finding;  2) Sample selection ambiguous and sample unlikely to be representative. | 1) Sample size estimation unclear or only sub-sample studied. | 1) Low response rate (<70%);  2) Response rate not reported. | 1) Assessment from non-validated data or generic estimate from the overall population. | 1) Data confusing. |

#

# eTable 3. Excluded studies with reasons

| **Excluded Studies** | **Reason for exclusion** |
| --- | --- |
| Valvano, et al. 2022^[1](#_ENREF_1)^ | Review |
| Tokar, et al. 2019^[2](#_ENREF_2)^ | No outcome data |
| Telford, et al. 2022^[3](#_ENREF_3)^ | No outcome data |
| Shibuya, et al. 2017^[4](#_ENREF_4)^ | No specific antiplatelet drugs data |
| Shalman, et al. 2015^[5](#_ENREF_5)^ | Review |
| Rodríguez de Santiago, 2022^[6](#_ENREF_6)^ | No specific antiplatelet drugs data |
| Manocha, et al. 2012^[7](#_ENREF_7)^ | No specific antiplatelet drugs data |
| Khubchandani, et al. 2011^[8](#_ENREF_8)^ | No outcome data |
| Jiang, et al. 2021^[9](#_ENREF_9)^ | No outcome data |
| Gerson, et al. 2010^[10](#_ENREF_10)^ | No outcome data |
| Gandhi, et al. 2013^[11](#_ENREF_11)^ | Review |
| Friedland, et al. 2006^[12](#_ENREF_12)^ | No outcome data |
| Blacker, et al. 2003^[13](#_ENREF_13)^ | No colonoscopy data |
| Assaad, et al. 2013^[14](#_ENREF_14)^ | No specific antiplatelet drugs data |
| Amato, et al. 2016^[15](#_ENREF_15)^ | No specific antiplatelet drugs data |
| Tsoi, et al. 2020^[16](#_ENREF_16)^ | No specific antiplatelet drugs data |
| Tani, et al. 2019^[17](#_ENREF_17)^ | No outcome data |
| Shimodate, et al. 2018^[18](#_ENREF_18)^ | No outcome data |

# eTable 4. Detailed demographics and clinical characteristics of the included studies

| **Study** | **Mean Age (y)** | **Female (%)** | **CHA2DS2-VASc**  **score** | **HAS-BLED score** | **CVD history**  **(%)** | **HBP**  **(%)** | **DM (%)** | **TIA history (%)** | **AF (%)** | **HF (%)** |
| --- | --- | --- | --- | --- | --- | --- | --- | --- | --- | --- |
| Yu, 2019 | 68.60 | 33.80 | 1.02 | NR | 21.80 | NR | NR | NR | NR | NR |
| Yao, 2020 | 62.10 | 32.50 | NR | NR | NR | 42.50 | 23.20 | NR | 2.50 | NR |
| Li, 2023 | 64.19 | 47.60 | 1.00 | NR | NR | 25.10 | NR | 5.10 | 6.40 | 4.80 |
| Yan, 2021 | 58.20 | 23.60 | NR | NR | NR | 41.20 | 14.30 | NR | NR | 16.50 |
| Yabe, 2021 | 69.00 | 39.00 | NR | NR | NR | NR | NR | NR | NR | NR |
| Makino, 2018 | 72.00 | 42.00 | NR | NR | NR | NR | NR | NR | NR | NR |
| Lin, 2018 | 64.00 | 0.00 | NR | NR | NR | 79.00 | 42.00 | NR | NR | 17.00 |
| Kishino, 2020 | 69.80 | 28.50 | NR | NR | 10.20 | NR | 18.90 | NR | NR | NR |
| Kishida, 2019 | 66.00 | 14.00 | NR | NR | NR | NR | NR | NR | NR | NR |
| Kim, 2021 | 68.90 | 22.80 | NR | NR | NR | 65.60 | 30.80 | NR | 45.60 | NR |
| Hayasaka, 2023 | 73.50 | 21.90 | NR | NR | NR | NR | NR | NR | NR | NR |
| Friedland, 2009 | 68.40 | 0.81 | NR | NR | NR | NR | NR | NR | NR | NR |
| Chan, 2019 | 62.50 | 17.80 | NR | NR | NR | NR | NR | NR | NR | NR |
| Bozkurt, 2021 | 65.30 | 29.00 | NR | NR | 33.00 | 52.10 | 26.60 | NR | NR | NR |
| Aizawa, 2022 | 72.70 | 23.40 | NR | NR | NR | NR | NR | NR | NR | NR |
| Singh, 2010 | 65.30 | 2.00 | NR | NR | 52.00 | 74.00 | 34.00 | NR | NR | NR |
| Feagins, 2011 | 63.20 | 3.56 | NR | NR | NR | 72.60 | 38.60 | NR | NR | NR |
| Pan, 2012 | 68.10 | 43.40 | NR | NR | NR | NR | NR | NR | NR | NR |
| Yousfi M, 2004 | 72.00 | 48.30 | NR | NR | NR | NR | NR | NR | NR | NR |
| Feagins, 2013 | 62.40 | 3.00 | NR | NR | NR | 72.00 | 35.23 | NR | NR | 10.20 |
| Amato, 2016 | 59.00 | 45.70 | NR | NR | NR | NR | NR | NR | NR | NR |
| Matsumoto, 2018 | 61.80 | 25.00 | NR | NR | NR | NR | NR | NR | NR | NR |

DM: Diabetes; HF: heart failure; HBP: hypertension; TIA: transient ischemic attack; CVD: cardiovascular disease; NR: not reported.

#

# eTable 5. Quality scores of the included studies

| **Study** | **Sample population** | **Sample size** | **Participation rate** | **Outcomeassessment** | **Analytical methods to control for bias** | **Total score** |
| --- | --- | --- | --- | --- | --- | --- |
| Yousfi M, 2004 | 2 | 1 | 1 | 1 | 1 | 6 |
| Friedland, 2009 | 2 | 2 | 2 | 1 | 2 | 9 |
| Singh, 2010 | 2 | 2 | 2 | 1 | 2 | 9 |
| Feagins, 2011 | 2 | 2 | 2 | 1 | 1 | 8 |
| Pan, 2012 | 2 | 2 | 2 | 1 | 2 | 9 |
| Feagins, 2013 | 2 | 2 | 2 | 1 | 1 | 8 |
| Amato, 2016 | 2 | 2 | 2 | 1 | 2 | 9 |
| Lin, 2018 | 2 | 2 | 1 | 1 | 1 | 7 |
| Makino, 2018 | 2 | 1 | 1 | 1 | 1 | 6 |
| Matsumoto, 2018 | 2 | 2 | 2 | 1 | 1 | 8 |
| Chan, 2019 | 2 | 2 | 2 | 1 | 2 | 9 |
| Kishida, 2019 | 2 | 2 | 2 | 1 | 1 | 8 |
| Yu, 2019 | 2 | 2 | 2 | 1 | 2 | 9 |
| Kishino, 2020 | 2 | 2 | 2 | 1 | 1 | 8 |
| Yao*,* 2020 | 2 | 2 | 2 | 1 | 2 | 9 |
| Bozkurt*,* 2021 | 2 | 2 | 2 | 1 | 1 | 8 |
| Kim, 2021 | 2 | 2 | 2 | 1 | 2 | 9 |
| Yabe, 2021 | 2 | 2 | 2 | 1 | 1 | 8 |
| Yan, 2021 | 2 | 2 | 2 | 1 | 2 | 9 |
| Aizawa, 2022 | 2 | 2 | 2 | 1 | 1 | 8 |
| Hayasaka, 2023 | 2 | 2 | 2 | 1 | 1 | 8 |
| Li, 2023 | 2 | 2 | 2 | 1 | 2 | 9 |

# eTable 6. Sensitivity analysis of PPB/TE rate in patients with or without continued clopidogrel therapy

| **Sensitivity analysis of PPB rate in patients with continued clopidogrel therapy** | | | | | |
| --- | --- | --- | --- | --- | --- |
| **Study omitted** | **PPB rate (95%CI)** | **Study omitted** | **Prevalence (95%CI)** | **Study omitted** | **Prevalence (95%CI)** |
| Yu, 2019 | 0.034(0.016-0.052) | Yan, 2021 | 0.023(0.012-0.034) | Makino, 2018 | 0.025(0.013-0.037) |
| Li, 2023 | 0.024(0.013-0.035) | Yabe, 2021 | 0.023(0.012-0.035) | Lin, 2018 | 0.034(0.016-0.051) |
| Chan, 2019 | 0.023(0.012-0.034) | Feagins, 2011 | 0.028(0.016-0.041) | Singh, 2010 | 0.021(0.011-0.032) |
| Feagins, 2013 | 0.015(0.008-0.023) |  |  |  |  |
| **Sensitivity analysis of PPB rate in patients without continued clopidogrel therapy** | | | | | |
| Kim, 2021 | 0.020(0.000-0.041) | Singh, 2010 | 0.018(0.000-0.037) | Feagins, 2011 | 0.030(0.022-0.038) |
| Chan, 2019 | 0.020(0.000-0.039) | Bozkurt, 2021 | 0.023(0.002-0.043) |  |  |
| **Sensitivity analysis of TE rate in patients with continued clopidogrel therapy** | | | | | |
| Yu, 2019 | 0.003(0.002-0.004) | Li, 2023 | 0.028(0.000-0.059) |  |  |

CI: confidence interval; PPB: post-polypectomy bleeding; TE: thromboembolism.

# eTable 7. Sensitivity analysis of PPB rate in patients with or without continued aspirin therapy

| **Sensitivity analysis of PPB rate in patients with continued aspirin therapy** | | | | | |
| --- | --- | --- | --- | --- | --- |
| **Study omitted** | **PPB rate (95%CI)** | **Study omitted** | **Prevalence (95%CI)** | **Study omitted** | **Prevalence (95%CI)** |
| Yao, 2020 | 0.017(0.010-0.024) | Yan, 2021 | 0.018(0.011-0.025) | Lin, 2018 | 0.021(0.013-0.029) |
| Li, 2023 | 0.017(0.010-0.024) | Yabe, 2021 | 0.021(0.012-0.031) | Hui, 2004 | 0.017(0.010-0.024) |
| Bozkurt, 2021 | 0.017(0.010-0.024) | Pan, 2012 | 0.018(0.010-0.025) | Amato, 2016 | 0.017(0.011-0.024) |
| Aizawa, 2022 | 0.016(0.009-0.023) | Yousfi M, 2004 | 0.014(0.008-0.020) | Matsumoto, 2018 | 0.017(0.010-0.024) |
| **Sensitivity analysis of PPB rate in patients without continued aspirin therapy** | | | | | |
| Li, 2023 | 0.012(0.005-0.019) | Hayasaka, 2023 | 0.014(0.005-0.022) | Pan, 2012 | 0.018(0.009-0.026) |
| Kishino, 2020 | 0.015(0.006-0.024) |  |  |  |  |

CI: confidence interval; PPB: post-polypectomy bleeding.

# eTable 8. Meta-regression of the risk of bleeding and thromboembolic events in patients undergoing colonoscopy.

| **Variables** | **No. of reported studies** | **β coefficient (95%CI)** | ***P* value** |
| --- | --- | --- | --- |
| Mean age | 22 | 1.006 (0.9762-1.0370) | 0.658 |
| Female | 22 | 0.9978 (0.9891-1.0065) | 0.579 |
| HF | 5 | 1.005 (0.5409-1.8672) | 0.935 |
| HBP | 9 | 0.0093 (0.9711-1.0302) | 0.984 |
| DM | 9 | 1.0010 (0.9570-1.0472) | 0.946 |
| CHA2DS2-VASc score | 5 | 0.7524 (0.0047-1.5165) | 0.607 |
| HAS-BLED score | 5 | 1.0768 (0.4418-2.6243) | 0.755 |
| CVD history | 5 | 0.9977 (0.7844-1.2690) | 0.924 |
| TIA history | 5 | 0.7232 (0.0087-1.2510) | 0.523 |
| AF | 5 | 1.0203 (0.7152-1.4554) | 0.604 |

DM: Diabetes; HF: heart failure; HBP: hypertension; TIA: transient ischemic attack; CVD: cardiovascular disease; HF: heart failure; AF: atrial fibrillation.

**
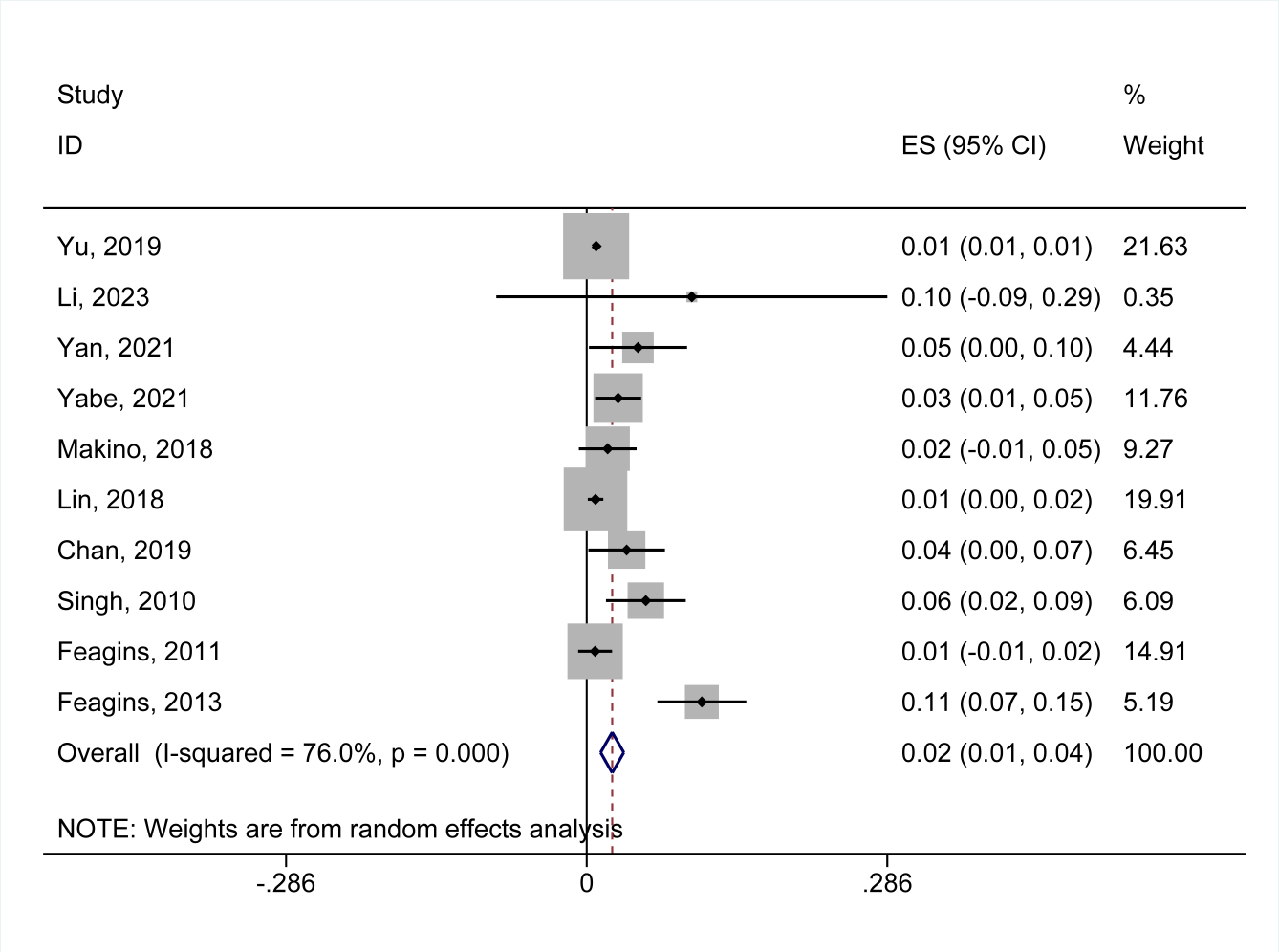
**

**eFigure 1. Pooled prevalence of PPB rate in patients with continued clopidogrel therapy**

**
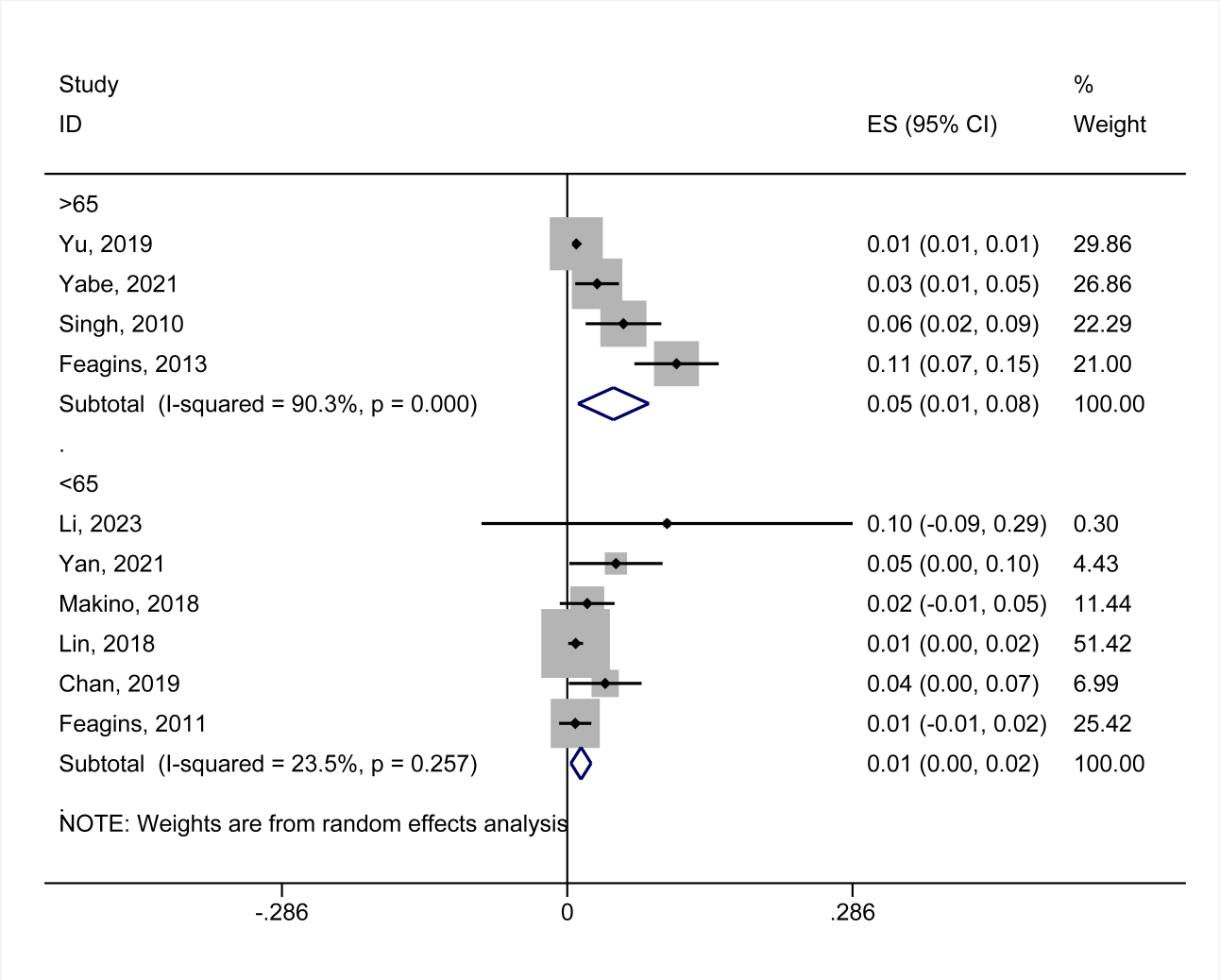
**

**eFigure 2. Pooled prevalence of PPB rate in patients with continued clopidogrel by age**

**
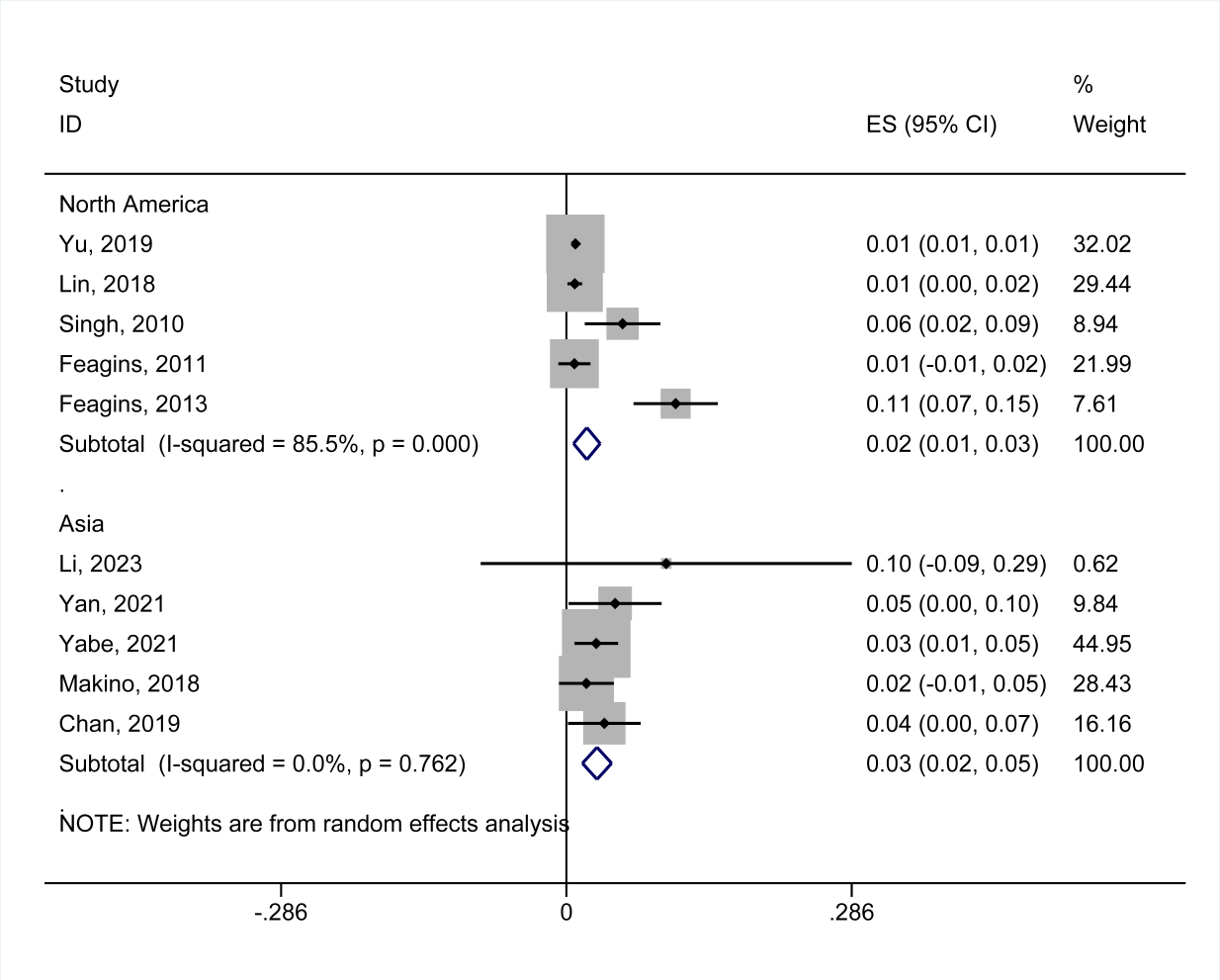
**

**eFigure 3. Pooled prevalence of PPB in patients with continued clopidogrel by region**

**
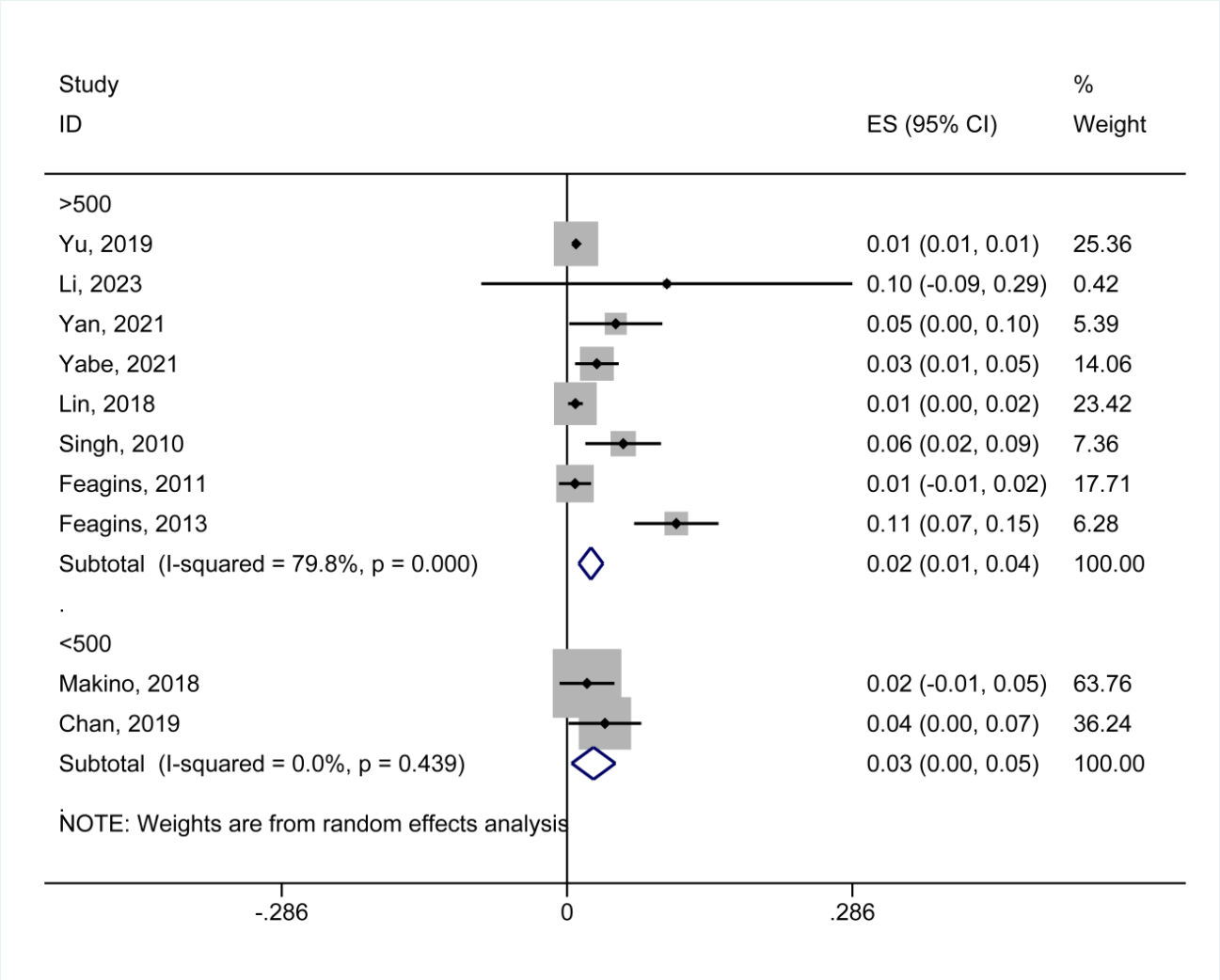
**

**eFigure 4. Pooled prevalence of PPB in patients with continued clopidogrel by sample**

**

**

**eFigure 5. Pooled prevalence of PPB rate in patients without continued clopidogrel**

**
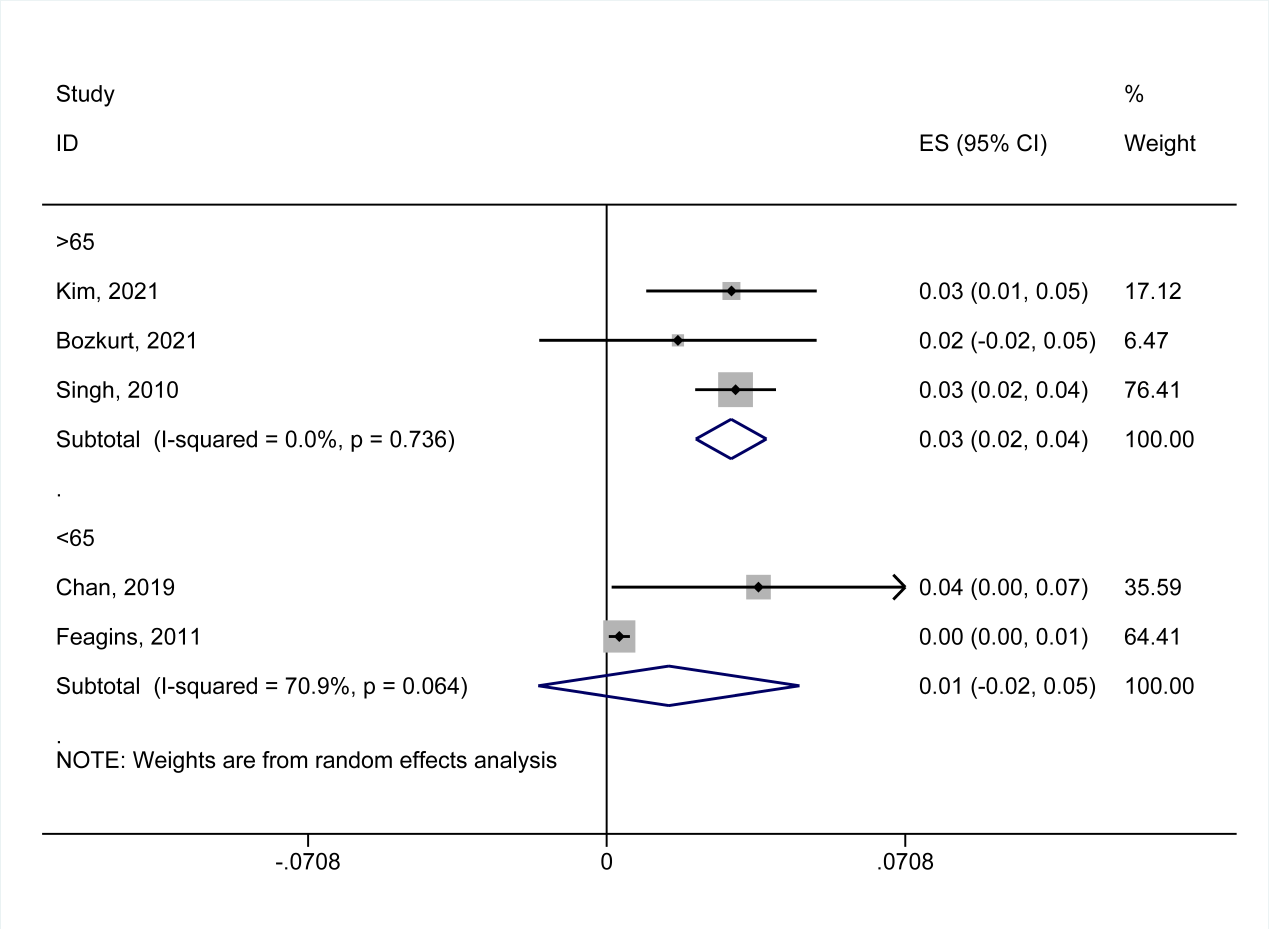
**

**eFigure 6. Pooled prevalence of PPB in patients without continued clopidogrel by age**

**

eFigure 7. Pooled prevalence of PPB in patients without continued clopidogrel by region**

**
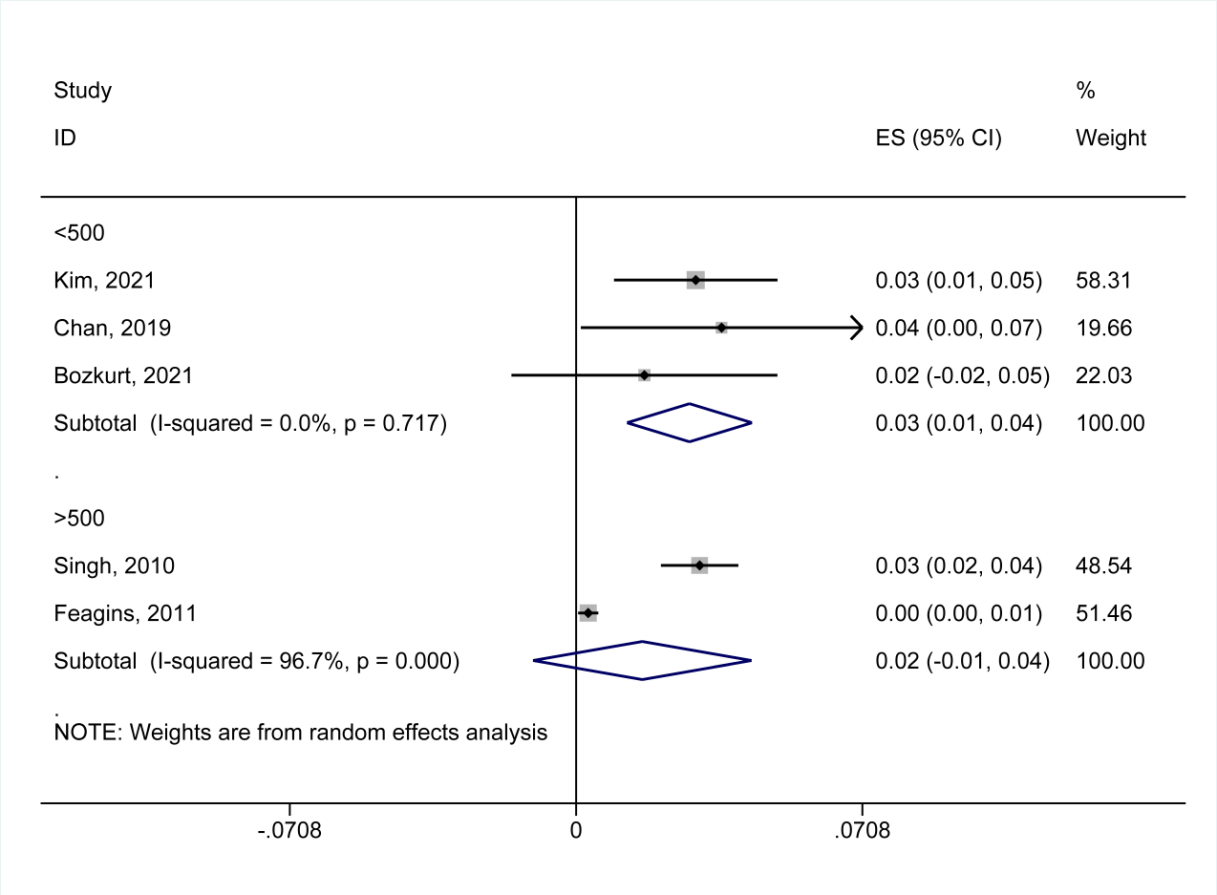
**

**eFigure 8. Pooled prevalence of PPB in patients without continued clopidogrel by sample**

**
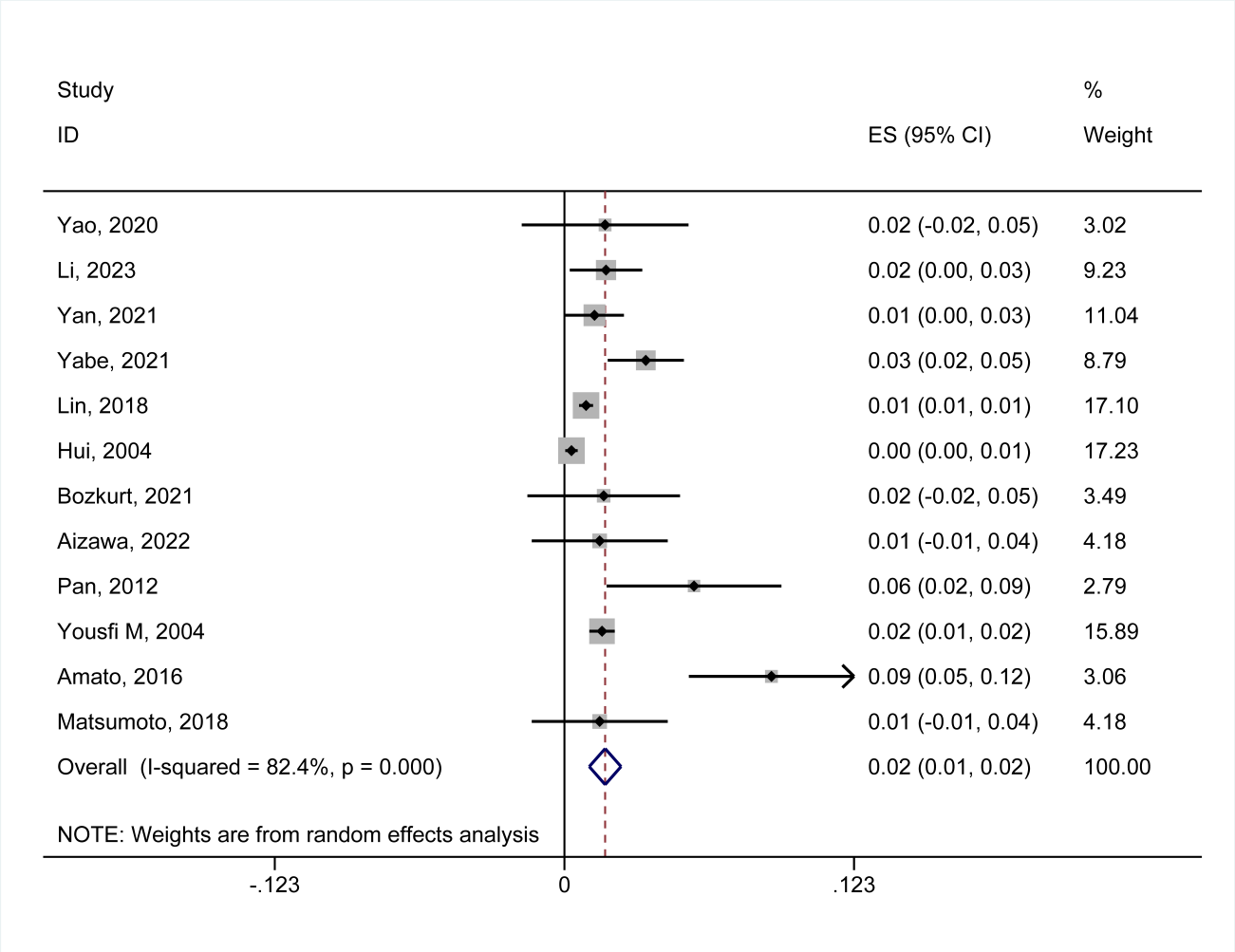
**

**eFigure 9.** **Pooled prevalence of PPB rate in patients with continued aspirin therapy**

**
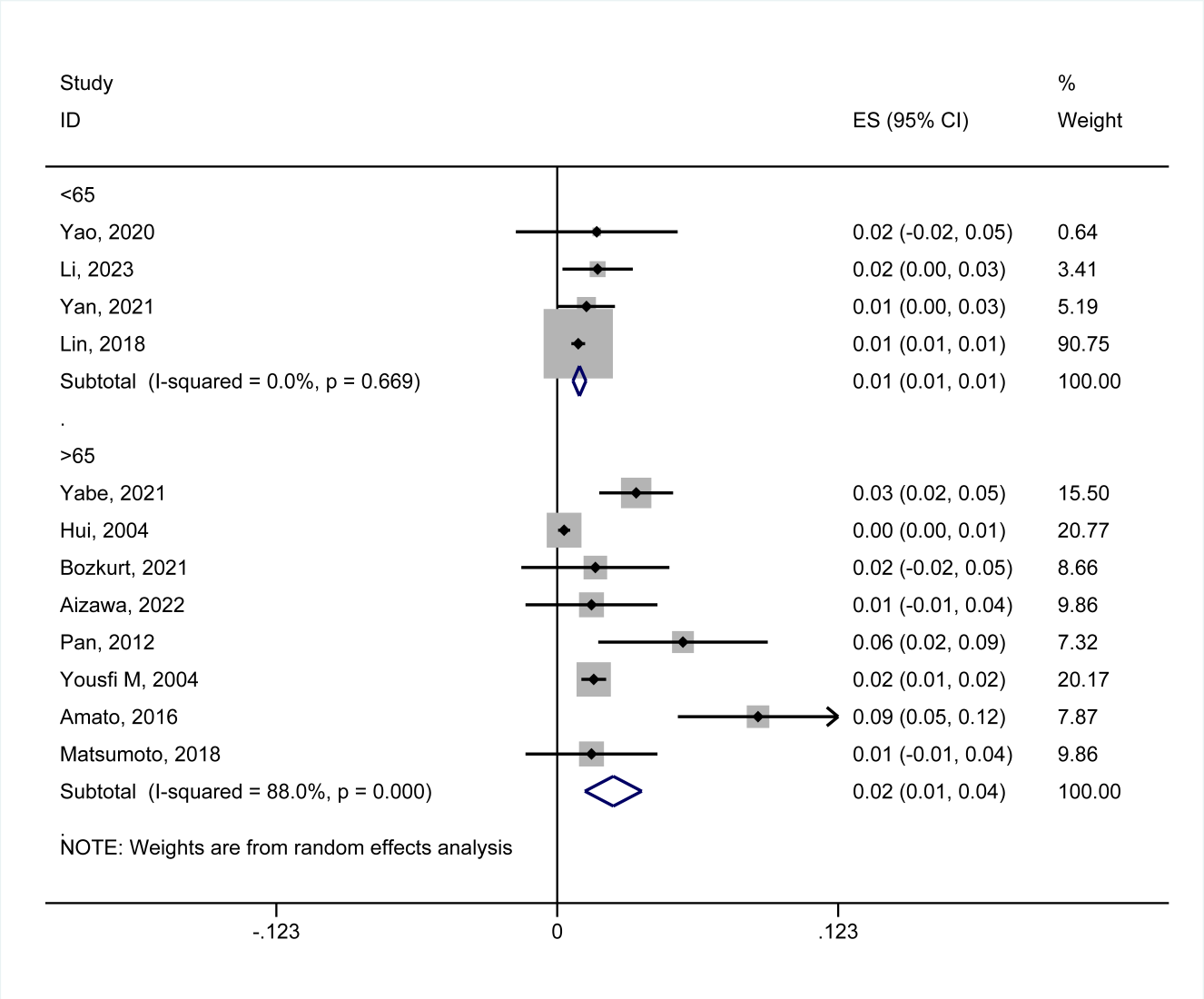
**

**eFigure 10. Pooled prevalence of PPB rate in patients with continued aspirin by age**

**
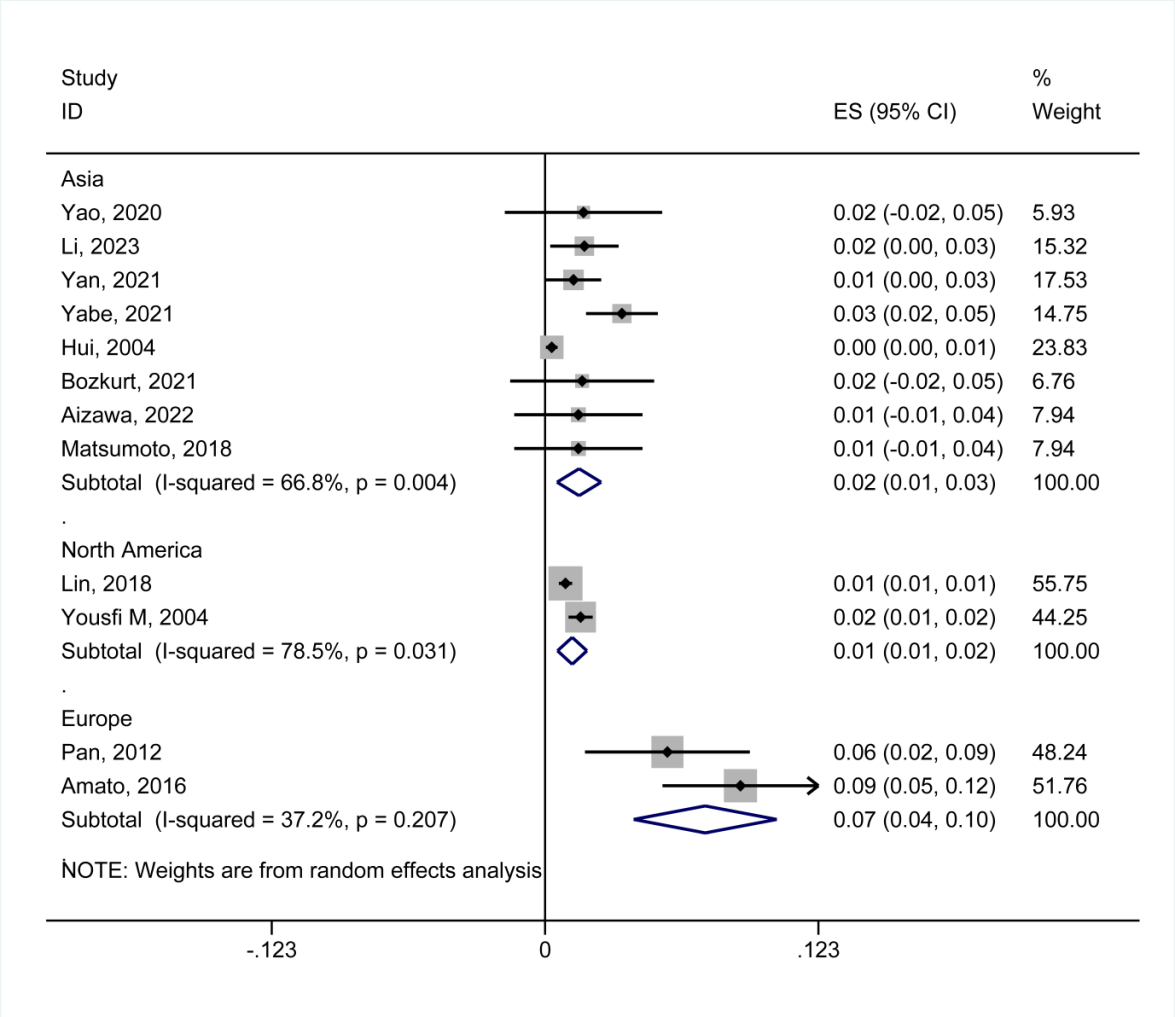
**

**eFigure 11. Pooled prevalence of PPB in patients with continued aspirin by region**

**
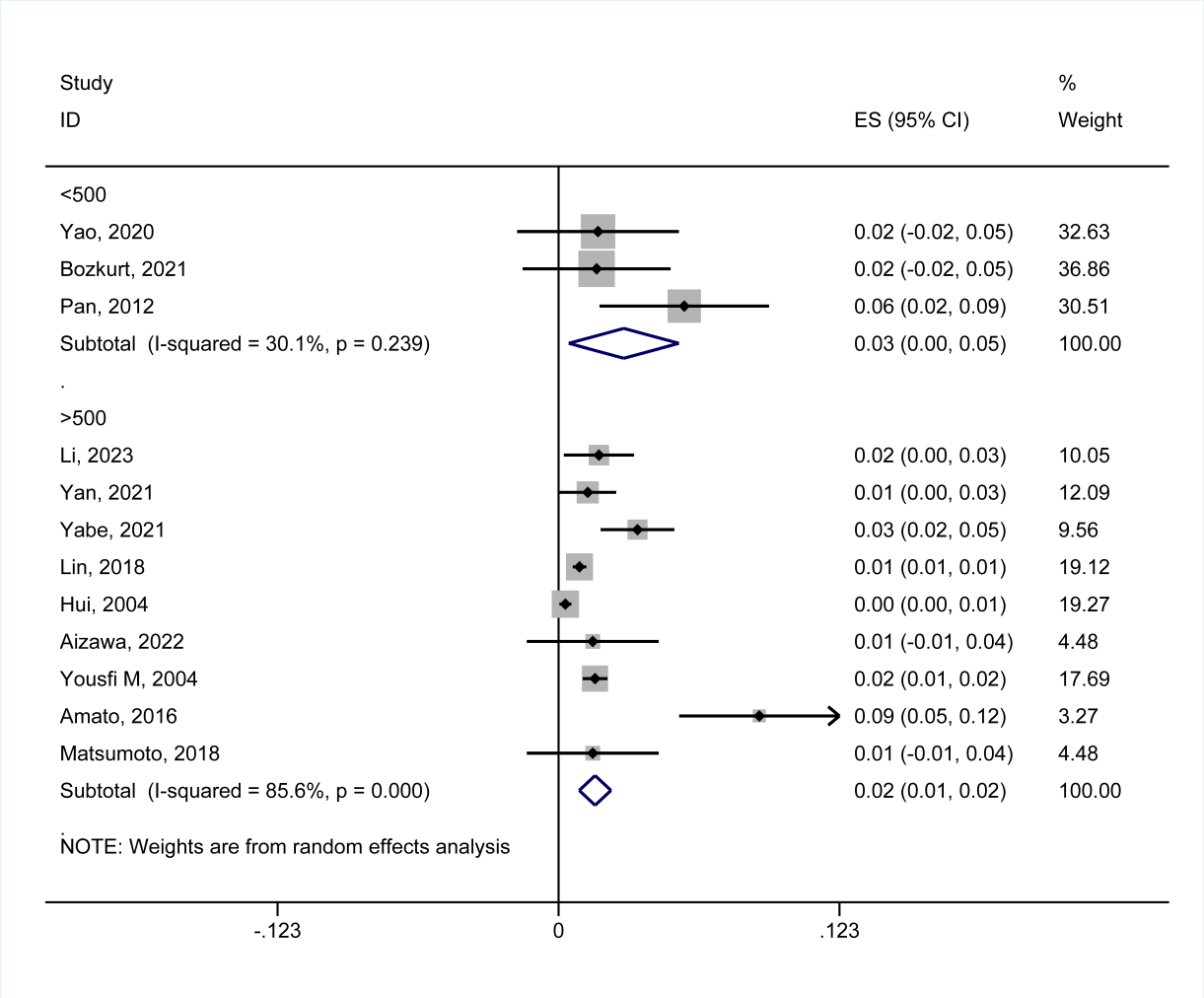
**

**eFigure 12. Pooled prevalence of PPB in patients with continued aspirin by sample**

**

**

**eFigure 13. Pooled prevalence of PPB rate in patients without continued aspirin therapy**

**
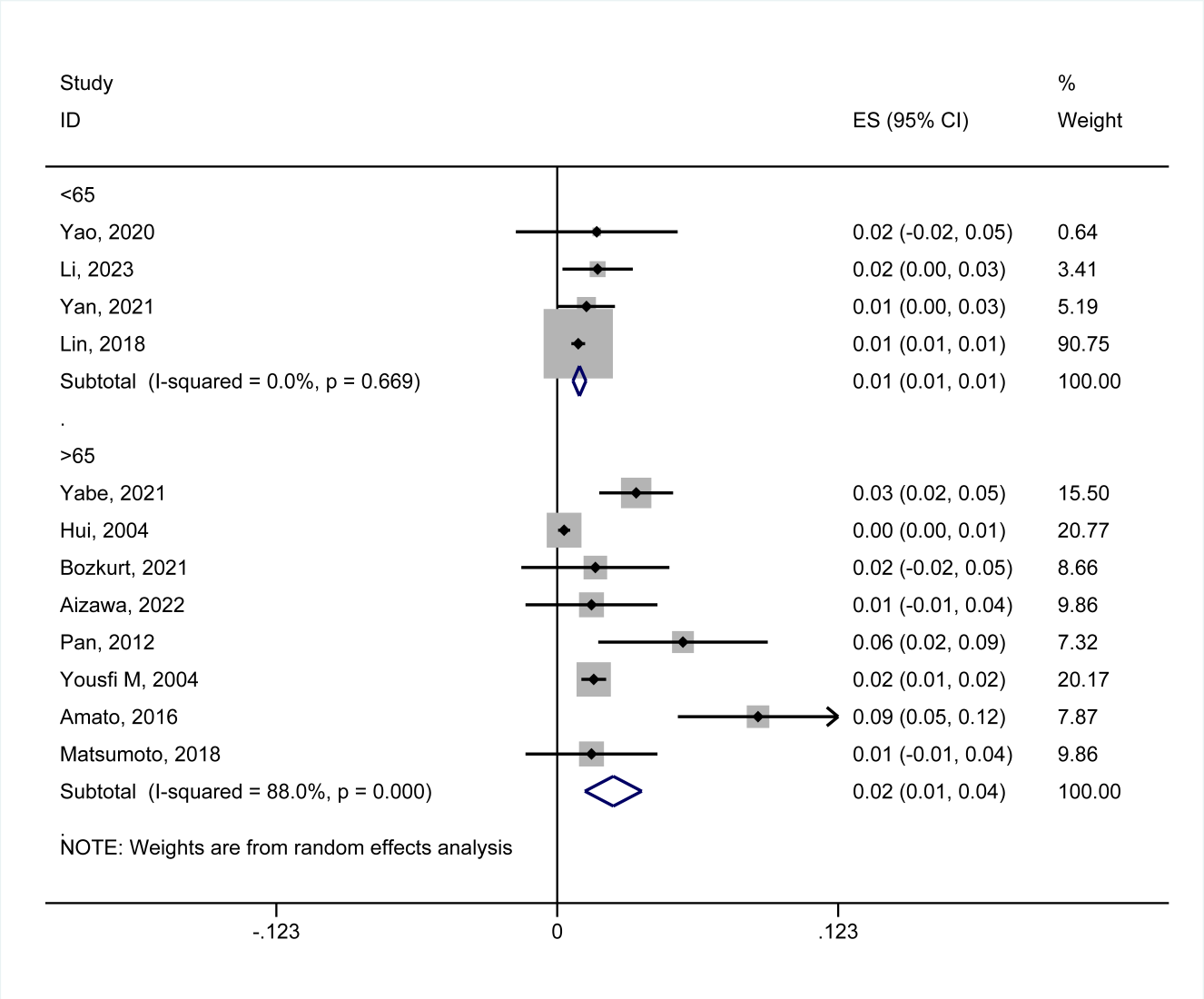
**

**eFigure 14. Pooled prevalence of PPB rate in patients with continued aspirin by age**

**
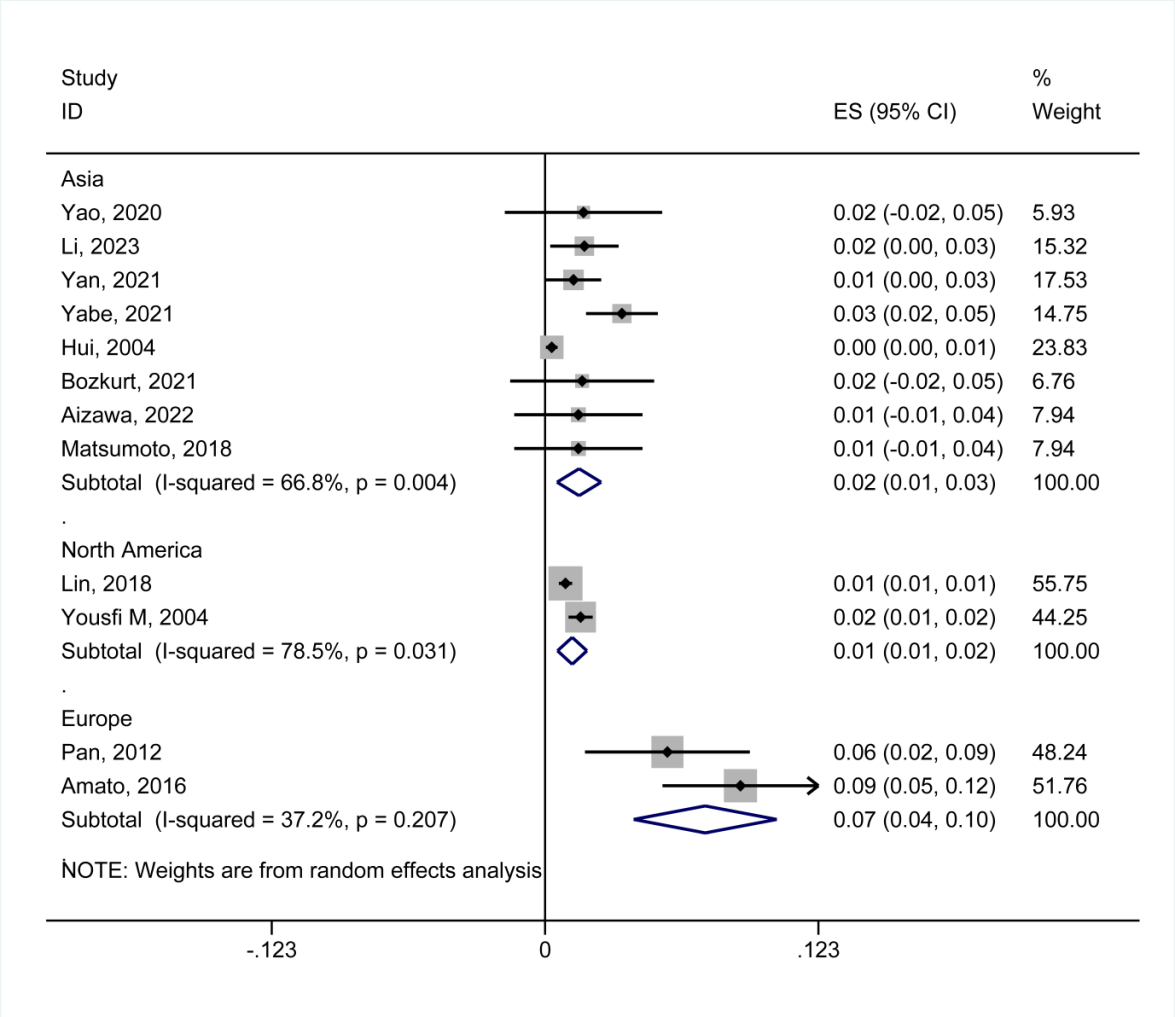
**

**eFigure 15. Pooled prevalence of PPB in patients with continued aspirin by region**

**
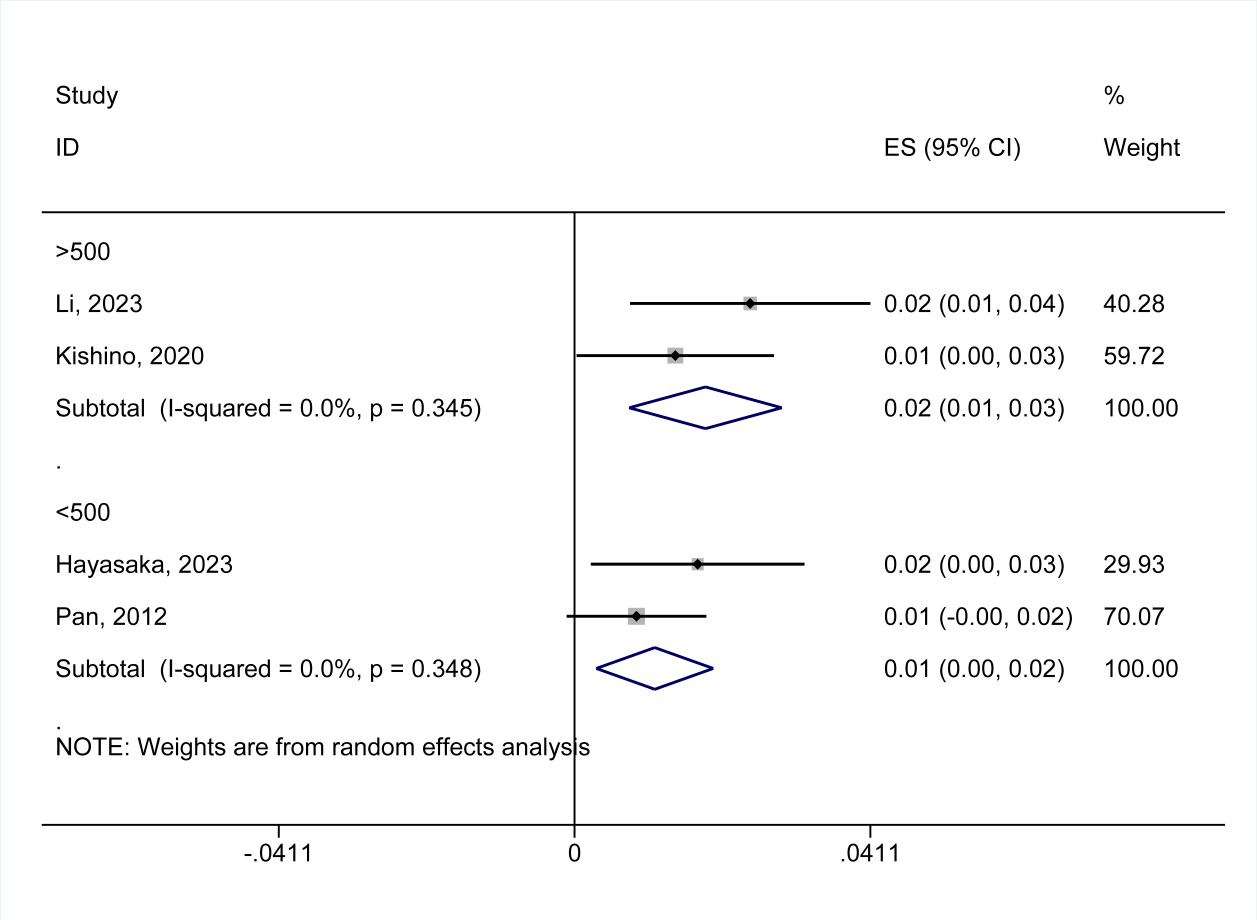
**

**eFigure 16. Pooled prevalence of PPB in patients without continued aspirin by sample**

**

**

**eFigure 17. Pooled prevalence of TE rate in patients with continued clopidogrel therapy**

**
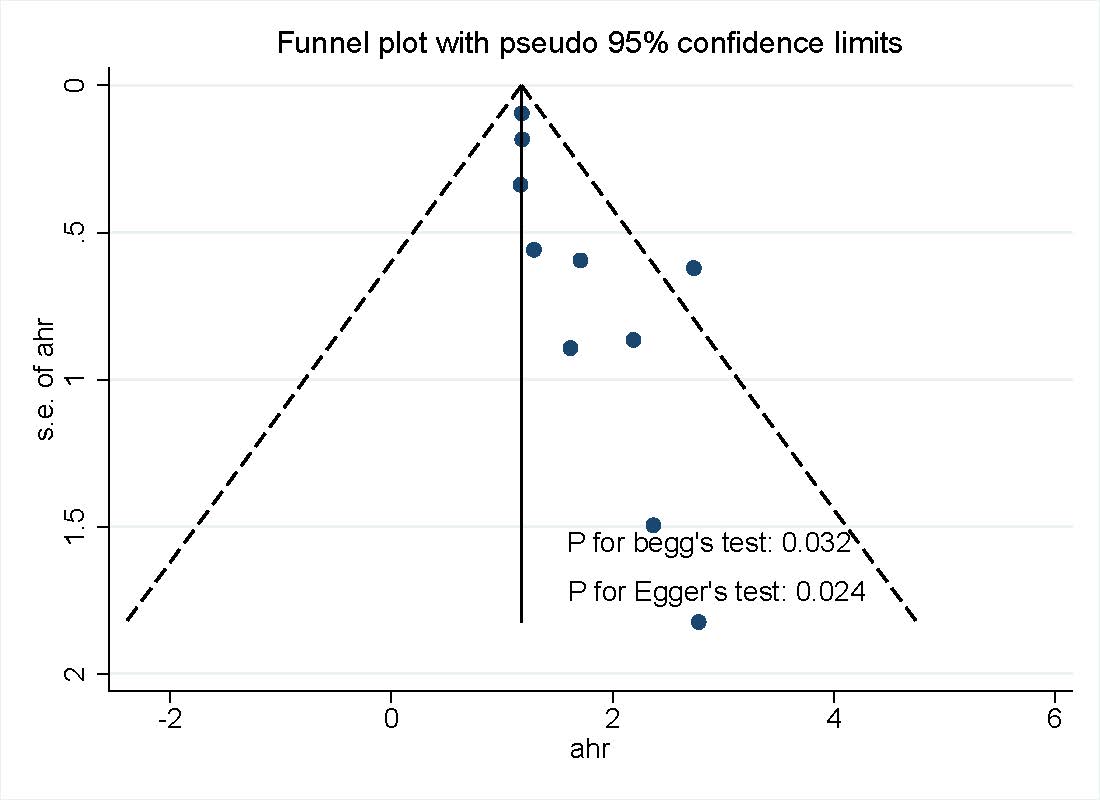
**

# eFigure18. Publication bias of studies on PPB rate in patients with continued clopidogrel therapy


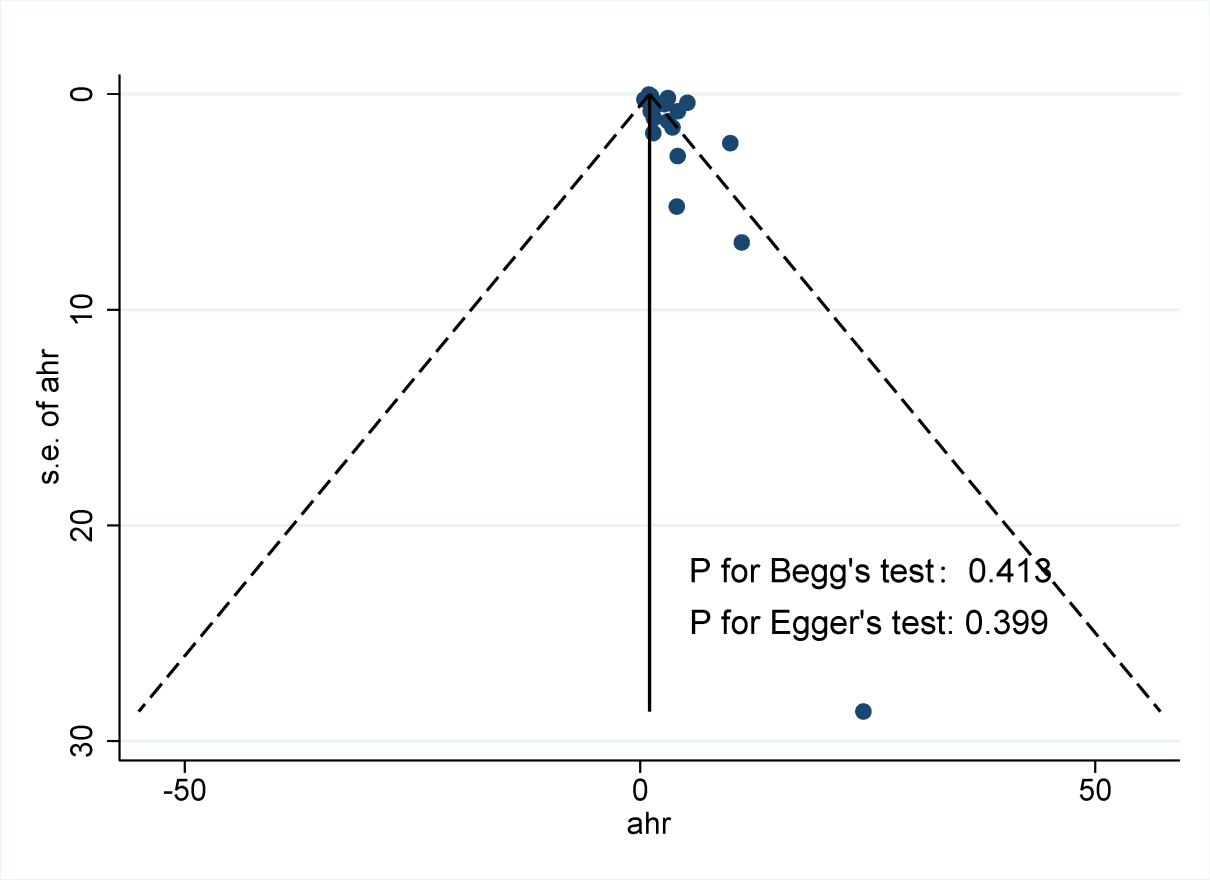


**eFigure19. Publication bias of studies on PPB rate in patients with continued**

**aspirin therapy**

# References

1. Valvano M, Fabiani S, Magistroni M, Mancusi A, Longo S, Stefanelli G, Vernia F, Viscido A, Romano S, Latella G. Risk of colonoscopic post-polypectomy bleeding in patients on single antiplatelet therapy: Systematic review with meta-analysis. 2022;36:2258-2270

2. Tokar JL, Bartel MJ. Colonoscopic polypectomy in patients receiving anticoagulation therapy: Some like it cold. *Annals of internal medicine*. 2019;171:285-286

3. Telford JJ, Abraham NS. Management of antiplatelet and anticoagulant agents before and after polypectomy. *Gastrointestinal endoscopy clinics of North America*. 2022;32:299-312

4. Shibuya T, Nomura O, Kodani T, Murakami T, Fukushima H, Tajima Y, Matsumoto K, Ritsuno H, Ueyama H, Inami Y, Ishikawa D, Matsumoto K, Sakamoto N, Osada T, Nagahara A, Ogihara T, Watanabe S. Continuation of antithrombotic therapy may be associated with a high incidence of colonic post-polypectomy bleeding. *Digestive endoscopy : official journal of the Japan Gastroenterological Endoscopy Society*. 2017;29:314-321

5. Shalman D, Gerson LB. Systematic review with meta-analysis: The risk of gastrointestinal haemorrhage post-polypectomy in patients receiving anti-platelet, anti-coagulant and/or thienopyridine medications. *Alimentary pharmacology & therapeutics*. 2015;42:949-956

6. Rodríguez de Santiago E, Sánchez Aldehuelo R, Riu Pons F, Rodríguez Escaja C, Fernández-Esparrach G, Cañete-Ruiz Á, Ferre Aracil C, Pérez-Corte D, Ríos León R, Marcos-Prieto HM, Delgado-Guillena PG, García-Rodríguez A, Guarner-Argente C, Muriel A, de la Fuente-Briongos E, García García de Paredes A, Parejo-Carbonell S, Téllez L, Senosiaín-Lalastra C, Burgos-Santamaría D, Aicart-Ramos M, Mateos Muñoz B, Peñas-García B, Pagano G, Casals Urquiza G, Urpi Ferreruela M, Ángel de Jorge-Turrión M, Barreiro-Alonso E, Fraile-López M, Gómez-Outomuro A, Altamirano MI, Núñez Esteban M, Ruiz-Andreu M, Arribas-Anta J, de Frutos D, Herreros-de-Tejada A, Arias-Rivera ML, Roldán-Fernández M, Marcos Martín Á F, Zamora J, Vázquez-Sequeiros E, Albillos A. Endoscopy-related bleeding and thromboembolic events in patients on direct oral anticoagulants or vitamin k antagonists. *Clinical gastroenterology and hepatology : the official clinical practice journal of the American Gastroenterological Association*. 2022;20:e380-e397

7. Manocha D, Singh M, Mehta N, Murthy UK. Bleeding risk after invasive procedures in aspirin/nsaid users: Polypectomy study in veterans. *The American journal of medicine*. 2012;125:1222-1227

8. Khubchandani IT, Heyrosa MG, Thekkeurumbil SV. Optimal timing of anticoagulation pre- and post-colonoscopy with polypectomy. *Techniques in coloproctology*. 2011;15:185-189

9. Jiang W, Suen BY, Ho HT, Ching JYL, Chan FKL, Mak JWY. Impact of physicians' and patients' compliance on outcomes of colonoscopic polypectomy with anti-thrombotic therapy. *Clinical gastroenterology and hepatology : the official clinical practice journal of the American Gastroenterological Association*. 2021;19:2559-2566.e2551

10. Gerson LB, Michaels L, Ullah N, Gage B, Williams L. Adverse events associated with anticoagulation therapy in the periendoscopic period. *Gastrointestinal endoscopy*. 2010;71:1211-1217.e1212

11. Gandhi S, Narula N, Mosleh W, Marshall JK, Farkouh M. Meta-analysis: Colonoscopic post-polypectomy bleeding in patients on continued clopidogrel therapy. *Alimentary pharmacology & therapeutics*. 2013;37:947-952

12. Friedland S, Soetikno R. Colonoscopy with polypectomy in anticoagulated patients. *Gastrointestinal endoscopy*. 2006;64:98-100

13. Blacker DJ, Wijdicks EF, McClelland RL. Stroke risk in anticoagulated patients with atrial fibrillation undergoing endoscopy. *Neurology*. 2003;61:964-968

14. Assaad B, Sesi VK, Figari R, Schultz L, Thummala N, Rehman M, Chandok A, Silverman A, Silver B. Antithrombotic management of stroke patients before colonoscopy. *Journal of stroke and cerebrovascular diseases : the official journal of National Stroke Association*. 2013;22:733-736

15. Amato A, Radaelli F, Dinelli M, Crosta C, Cengia G, Beretta P, Devani M, Lochis D, Manes G, Fini L, Paggi S, Passoni GR, Repici A. Early and delayed complications of polypectomy in a community setting: The spoc prospective multicentre trial. *Journal of Crohn's & colitis*. 2016;48:43-48

16. Tsoi A, Garg M, Butt J. Post-colonic polypectomy bleeding in patients on anticoagulation therapy. *Journal of Gastroenterology and Hepatology (Australia)*. 2020;35:232

17. Tani Y, Sakurai H, Nakamura T, Miyake M, Matsueda K, Ishihara R, Vanaclocha-Espi M, Ibáñez J, Molina-Barceló A, Valverde-Roig MJ, Pérez E, Nolasco A, de la Vega M, de la Lastra-Bosch ID, Oceja ME, Espinàs JA, Font R, Pérez-Riquelme F, Arana-Arri E, Portillo I, Salas D. Risk factors for severe complications of colonoscopy in screening programs. *Digestive endoscopy : official journal of the Japan Gastroenterological Endoscopy Society*. 2019;118:304-308

18. Shimodate Y, Mizuno M, Miyake M, Takezawa R, Doi A, Nishimura N, Mouri H, Matsueda K, Yamamoto H. Warfarin or direct oral anticoagulant; which is risky for post-polypectomy bleeding? *United European gastroenterology journal*. 2018;6:A182-A183
